# The role of ubiquitination in tumorigenesis and targeted drug discovery

**DOI:** 10.1038/s41392-020-0107-0

**Published:** 2020-02-29

**Authors:** Lu Deng, Tong Meng, Lei Chen, Wenyi Wei, Ping Wang

**Affiliations:** 1 College of Animal Science and Technology, Northwest A&F University, Yangling Shaanxi, 712100 China; 2 Division of Spine, Department of Orthopedics, Tongji Hospital Affiliated to Tongji University School of Medicine, 389 Xincun Road, Shanghai, China; 3 Division of Laboratory Safety and Services, Northwest A&F University, Yangling Shaanxi, 712100 China; 4 Department of Pathology, Beth Israel Deaconess Medical Center, Harvard Medical School, Boston, MA 02215 USA; 5 Tongji University Cancer Center, Shanghai Tenth People’s Hospital of Tongji University, School of Medicine, Tongji University, Shanghai, 200092 China

**Keywords:** Cancer metabolism, Cancer therapy, Cancer microenvironment, Cancer stem cells

## Abstract

Ubiquitination, an important type of protein posttranslational modification (PTM), plays a crucial role in controlling substrate degradation and subsequently mediates the “quantity” and “quality” of various proteins, serving to ensure cell homeostasis and guarantee life activities. The regulation of ubiquitination is multifaceted and works not only at the transcriptional and posttranslational levels (phosphorylation, acetylation, methylation, etc.) but also at the protein level (activators or repressors). When regulatory mechanisms are aberrant, the altered biological processes may subsequently induce serious human diseases, especially various types of cancer. In tumorigenesis, the altered biological processes involve tumor metabolism, the immunological tumor microenvironment (TME), cancer stem cell (CSC) stemness and so on. With regard to tumor metabolism, the ubiquitination of some key proteins such as RagA, mTOR, PTEN, AKT, c-Myc and P53 significantly regulates the activity of the mTORC1, AMPK and PTEN-AKT signaling pathways. In addition, ubiquitination in the TLR, RLR and STING-dependent signaling pathways also modulates the TME. Moreover, the ubiquitination of core stem cell regulator triplets (Nanog, Oct4 and Sox2) and members of the Wnt and Hippo-YAP signaling pathways participates in the maintenance of CSC stemness. Based on the altered components, including the proteasome, E3 ligases, E1, E2 and deubiquitinases (DUBs), many molecular targeted drugs have been developed to combat cancer. Among them, small molecule inhibitors targeting the proteasome, such as bortezomib, carfilzomib, oprozomib and ixazomib, have achieved tangible success. In addition, MLN7243 and MLN4924 (targeting the E1 enzyme), Leucettamol A and CC0651 (targeting the E2 enzyme), nutlin and MI‐219 (targeting the E3 enzyme), and compounds G5 and F6 (targeting DUB activity) have also shown potential in preclinical cancer treatment. In this review, we summarize the latest progress in understanding the substrates for ubiquitination and their special functions in tumor metabolism regulation, TME modulation and CSC stemness maintenance. Moreover, potential therapeutic targets for cancer are reviewed, as are the therapeutic effects of targeted drugs.

## Introduction

Ubiquitin (Ub), a highly conserved regulatory protein containing 76 amino acids, can be covalently tagged to target proteins via a cascade of enzymatic reactions, including Ub-activating (E1), Ub-conjugating (E2) and Ub-ligating (E3) enzymes. Subsequently, mono- or polyubiquitination regulates the function of a large number of proteins in various physiological and/or pathological conditions.^1,2^ Polyubiquitin with different chain topologies and lengths linked to specific lysine residues on substrates is associated with different functional consequences.^3^ Moreover, the function of Ub ligases can also be reversed by deubiquitinases (DUBs), which are also critical for almost all cellular signaling pathways, such as the cell cycle, apoptosis, receptor downregulation and gene transcription, by removing Ub from substrate proteins.^4,5^

Proteins are the fundamental units in regulating cellular functions, and ubiquitination is the second most common posttranslational modification (PTM) for proteins, behind only phosphorylation.^6^ Thus, aberrant ubiquitination may lead to disease development and progression, especially cancer.^7^ Mounting evidence suggests that alterations in the activity of many E3 ligases are significantly associated with the etiology of human malignancies.^8^ Mutations of E3 ligases may result in the rapid degradation of tumor suppressors or, conversely, the lack of ubiquitination of oncogenic proteins.^9^ The pathological processes not only involve tumor metabolism regulation but also contribute to immunological tumor microenvironment (TME) modulation and cancer stem cell (CSC) stemness maintenance.^10^ Moreover, due to their high substrate specificity, E3 ligases and DUBs are promising potential therapeutic targets for cancer treatment. Currently, anti-cancer drugs targeting the proteasome, E3 and DUBs have been actively developed, and their therapeutic effects have been suggested by animal experiments and clinical trials.^11,12^ Here, we specifically summarize the mechanisms of the different components of the ubiquitin proteasome system (UPS), including E1, E2, E3, the proteasome and deubiquitinating enzymes, in mediating substrate ubiquitination/deubiquitination, highlight the unique functions of ubiquitination in tumorigenesis, including tumor metabolism regulation, immunological TME modulation and CSC stemness maintenance, and review potential therapeutic targets and the therapeutic effects of targeted drugs.

## The components and processes of the UPS

### Ub

Ub, named for its wide distribution in various types of cells among eukaryotes, was first identified by Gideon Goldstein et al. in 1975 and further confirmed over the next several decades.^13,14^ In the human genome, Ub is encoded by four genes, namely, *UBB*, *UBC*, *UBA52* and *RPS27A*. The *UBA52* and *RPS27A* genes encode single copy Ub, which is fused to the N-terminus of the ribosomal protein subunits L40 and S27a, respectively; the *UBB* and *UBC* genes encode polyubiquitin molecules that repeat the tandem 3 and 9 times, respectively. In cells, DUBs specifically cleave these fusion proteins to produce active Ub molecules. Occasionally, the monomeric Ub unit cannot be directly utilized by E1, E2 or E3. For example, PTEN-induced putative kinase 1 (PINK1)-mediated phosphorylation of Ser at position 65 of Ub is necessary for the ubiquitination of mitochondrial membrane proteins. Therefore, phosphorylation at Ser65 of Ub plays an important role in mitophagy.^15–18^ In addition to Ser65, Ub can also be phosphorylated at Thr7, Thr12, Thr14, Ser20, Ser57, Tyr59 and Thr66, and phosphorylated monoubiquitin and polyubiquitin chains may alter their recognition by E3 ligases or Ub-binding proteins.^19–22^ Additionally, the Ub molecule can also be modified by other PTMs. For instance, the acetylation of Ub at K6 and K48 inhibits the formation and elongation of Ub chains.^23,24^ These characteristics further complicate the Ub codes, including the length of the Ub chain, the degree of mixing and the state of the branch.

### Ubiquitination

In 1977, Goldknopf et al. discovered that intracellular histones could be modified by ubiquitination, and ubiquitination emerged as a new protein PTM. In 2004, the Royal Swedish Academy of Sciences awarded the Nobel Prize in Chemistry to three scientists, Aaron Ciechanover, Avram Hershko and Irwin Rose, for their significant contributions in the field of ubiquitination.

**Fig. 1:**
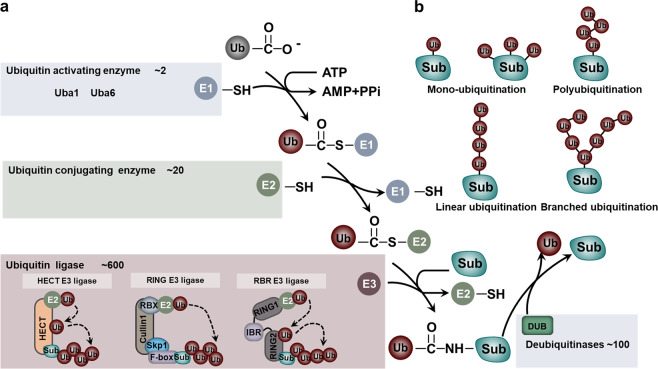
The components and processes of the UPS. **a** The components of the UPS and different classes of E3 ligases. **b** The ubiquitination linkage

Ubiquitination is carried out in a highly specific manner that labels substrate proteins with Ub. The attachment of Ub to the substrate requires an enzymatic cascade consisting of E1, E2 and E3.^13^ Specifically, these processes include a three-step enzymatic reaction. Initially, Ub is activated by E1 in an adenosine triphosphate-dependent manner and then is transferred to E2. This process involves the formation of a thioester bond between the active site Cys residue of E1 and the C-terminal carboxyl group of Ub (E1~Ub). The human genome encodes only two kinds of E1, namely, UBa1 and UBa6 (Fig. 1a).^25^ In the second step, E1 delivers the activated Ub to E2 and assists the specific E3s in transferring the activated Ub to the substrate. Generally, humans have 35 distinct Ub-binding enzymes. Although all E2s contain a very conserved Ub-binding catalytic domain, members of this family exhibit significant specificity in their interaction with E3s (Fig. 1a).^26,27^ Finally, E3 ligases catalyze the transfer of Ub from E2~Ub to a specific substrate protein. When this process is completed, an isopeptide bond is formed between the lysine ε-amino group of the substrate and the C-terminal carboxyl group of Ub (Fig. 1a). The E3 ligase is the largest and most complex component of the UPS.^26,28^ To date, more than 600 E3 Ub ligases have been identified in the human genome (Fig. 1a). Although some E2s can directly transfer Ub to substrate proteins, in most ubiquitination processes, substrate selection and Ub linkage are achieved by E3.^28,29^

### Ubiquitination linkage

According to the structural characteristics, three main types of ubiquitination linkages have been identified: monoubiquitination, polyubiquitination and branched ubiquitination (Fig. 1b). Monoubiquitination refers to the attachment of a single Ub to a specific lysine residue of the substrate under the enzymatic cascade of E1, E2 and E3.^5^ Accumulating evidence has revealed that monoubiquitination is involved in the regulation of DNA damage repair. For example, the E3 ligase Rad18 regulates proliferating cell nuclear antigen (PCNA) monoubiquitination in response to DNA damage repair via the recruitment of DNA polymerases.^30^ In addition, the monoubiquitination of H2AX driven by TNF receptor-associated factor 6 (TRAF6) is a prerequisite for recruiting ataxia telangiectasia mutated (ATM).^31^ In other cases, monoubiquitination does not regulate DNA damage repair but mediates other cellular processes, such as autophagy and chromatin remodeling. For instance, monoubiquitination of membrane proteins can modulate their interaction with the autophagy adapter protein p62, thereby promoting mitochondrial autophagy and peroxisome autophagy.^32^ In addition, a well-defined case is the lysine-specific monoubiquitination of histone, whose modification takes part in chromatin remodeling.^33^ Moreover, Ras can also undergo multiple ubiquitination events at multiple sites and then regulate various signaling pathways.^34^

Polyubiquitination refers to the attachment of more than two Ub molecules to the same lysine residue of the substrate. Compared to monoubiquitination, there are many types of polyubiquitination, which can be linked by any lysine residues in Ub (K6, K11, K27, K29, K33, K48 and K63) or through its N-terminal Met.^35,36^ Initially, the ubiquitination of the K48 type was considered to be the only polyubiquitination. It serves as a degradation signal for transferring proteins to the 26S proteasome.^37^ With the deepening of research, scientists have realized that K48-type polyubiquitination is only the tip of the ubiquitination iceberg.^23,38,39^ To date, eight types of polyubiquitin linkages have been identified (K6, K11, K27, K29, K33, K48, K63 and Met1) with specific functions. Unlike K48 polyubiquitination, K6 polyubiquitination takes part in the process of DNA damage repair;^40,41^ K11 polyubiquitination plays an important role in the cell cycle and trafficking events;^42,43^ K27 polyubiquitination regulates mitochondrial autophagy;^44,45^ K29 polyubiquitination modulates ubiquitin-fusion degradation (UFD)-mediated protein degradation;^46,47^ and K33 polyubiquitination participates in Toll receptor-mediated signaling pathways.^48,49^ K63 polyubiquitination typically takes part in protein–protein interactions, protein activity and trafficking, thereby regulating various biological processes.^50–52^ Moreover, Met1 is usually involved in the coupling of the C-terminus of Ub to the methionine (M1) residue on the substrate to form a peptide bond. This type of ubiquitination modification is catalyzed by a specific E3 ligase and usually controls the TNFα signaling pathway.^53–55^

A Ub chain with a single linkage is called a homologous chain, and a branched polyubiquitin chain contains a variety of linkages. To date, in addition to the polyubiquitination of K6, K11, K27, K29, K33, K48, K63 and Met1, branched polyubiquitination also plays an important role in regulating various cellular processes.^51^ For example, the mixed K11 and K63 linkages participate in the Epsin1-mediated endocytosis of major histocompatibility complex I (MHCI).^51,56^

### E3 ligases

E3 ligases are extraordinarily important in determining the specific type of ubiquitinated substrate. According to the catalytic structure, the E3 ligases are historically grouped into three types: the RING (really interesting new gene) family, the HECT (homologous to the E6-AP carboxyl terminus) family and the RBR (ring between ring fingers) family (Fig. 1a).^57^

#### RING type of E3 ligases

RING E3 is characterized by its RING or U-box folding catalytic domain that facilitates direct Ub transfer from E2 to the substrate (Fig. 1a). There are more than 600 E3 ligases in the human genome, and the RING family, encoded by ~270 human genes, is the largest family of E3 ligases. The RING finger protein generally contains the following amino acid sequence: Cys-X_2_-Cys-X_9–39_-Cys-X_1–3_-His-X_2–3_-Cys/His-X_2_-Cys-X_4–48_-Cys-X_2_-Cys, wherein X represents any amino acid. An E3 can bind directly to the substrate without the assistance of other proteins in catalyzing the ubiquitination of the substrate.^58,59^ For example, Mdm2 (murine double minute2)/Hdm2 (HDM2 being the human enzyme) and RNF152 (ring finger protein 152) belong to this class of E3s. The former promotes p53 degradation,^60^ and the latter mediates the polyubiquitination of RagA.^61^ Instead, some E3 catalytic domains and substrate recruitment modules are composed of multiple proteins, including SCF (Skp1-cullin1-F-box) and APC/C (anaphase promoting complex/cyclosome).^62,63^ SCF is a multisubunit complex consisting of four proteins: invariant Rbx1 (recruit the E2 enzyme), Cul1 (scaffold protein), Skp1 (bridge F-box proteins (FBPs)) and a different FBP (harbor catalytic activity). Approximately 70 FBPs have been identified in humans. Generally, the FBP takes effect in substrate recognition in the complex and selectively regulates many downstream biological processes.^64^ F-box/WD repeat-containing protein 7 (FBXW7) and S-phase kinase-associated protein 2 (SKP2) are well-studied FBPs. As an important tumor suppressor, FBXW7 participates in the degradation of many oncogenes, such as Myc, c-Jun, cyclin E, mTOR, Notch-1 and Mcl-1. Its mutation and deletion are often associated with tumorigenesis.^65^ SKP2, an important oncogene, regulates a number of CDK inhibitors (such as p27) and cell cycle proteins (such as p21, p57, cyclin A, cyclin E and cyclin D1).^66^ Another representative example is APC/C, which is the most sophisticated RING E3 ligase. APC/C contains a cullin-related scaffolding protein, APC2, to catalyze the ubiquitination reaction and further precisely controls cell cycle progression by alternately engaging with the substrate binding module, CDC20 (recruiting cell division cycle 20) or CDC20-like protein 1 (CDH1). APC/C-CDC20 promotes the cell cycle transition from metaphase to anaphase, while APC/C-CDH1 mediates mitotic exit and early G1 entry.^67,68^

#### HECT-type of E3 ligases

The second category of E3s is the HECT Ub ligase, which can be further divided into three subfamilies: Nedd4/Nedd4-like E3s containing a WW domain, HERC E3s containing an RLD domain and other E3s without a WW or RLD domain. Compared with RING E3s, there are fewer HECT E3s, with only 28 coding genes in the human genome.^59,69^ The most obvious feature of HECT E3s is the HECT domain, which forms a transiently covalent bound to Ub through a conserved Cys. Unlike RING E3s, HECT E3s bind to Ub in E2-Ub and form a thioester-linked intermediate before being ligated to the lysine residue of the substrate. That is, Ub is transferred from E2 to E3, and then E2 activates HECT, thereby linking Ub to the HECT E3s via a thioester bond and transferring Ub to the substrate (Fig. 1a).^70^

The polyubiquitination linkage promoted by HECT E3s is determined by the C-terminal region of E3s rather than E2s.^71^ For instance, E6-related protein (E6AP), the first identified HECT E3, promotes K48-linkage polyubiquitination and substrate degradation; Rsp5, which belongs to the Nedd4 family of HECT, adds K63 linkage polyubiquitination to the substrate and regulates cellular endocytosis (receptors, ion channels, etc.).^72,73^ It is surprising that replacing the 62 amino acid sequence in the C-terminal amino acid of Rsp5 with the corresponding sequence of E6AP makes Rsp5 form a specific K48 polyubiquitin chain.^74^ In addition to E6AP and Nedd4, HECT E3s bind directly to the PY motifs or variant regions of the substrate through the WW domain. This interaction is critical in regulating signaling pathways, especially in the Hippo and TGFβ signaling pathways.^75–78^

#### RBR-type of E3 ligases

The RBR family is a special type of E3 ligase with an activation mechanism that is different from those of the RING and HECT types. The human genome encodes more than a dozen RBR E3s, and the family members are all multidomain proteins consisting of really interesting new gene 1 (RING1), in-between RING (IBR) and really interesting new gene 2 (RING2) (Fig. 1a).^79^ Among them, RING1 binds to E2 and has the characteristics of RING-type E3s. RING2, which contains a catalytic Cys nucleophile, has a similar activity as HECT E3. It forms a thioester bond intermediate with Ub and transfers Ub to the substrate (Fig. 1a).^80–82^ The most striking E3 of the RBR family is the linear ubiquitin chain assembly complex (LUBAC) complex, consisting of HOIP, HOIL-1L and Sharpin. It is specifically responsible for regulating the linear ubiquitination of substrates, which plays a very important role in various biological processes, such as innate immunity and inflammation.^55,83–85^

All RBR E3s have a special regulation of self-inhibition due to their special structure. Mechanically, in the RBR E3 ligase, the domain outside the RING1, IBR and RING2 domains separates the RING2 domain from the RING1–IBR domain and structurally masks the active site Cys. The spatial distance between the active site of RING2 and E2 inhibits the thiol-transfer reaction and decreases the activity of RBR.^80,86,87^ Thus, the E3 ligase of the RBR family needs to undergo a conformational change to expose the Cys of RING2 and activate the E3 ligase.^86^

The activity of the RBR E3 family needs to be regulated in an orderly manner, and aberrant activity may lead to a number of diseases, including cancer and Parkinson’s disease (PD). For example, although its mutation is a major cause of familial PD,^88,89^ Parkin can function as a tumor suppressor to downregulate some substrates, such as cyclin D and cyclin E, and subsequently control cell cycle progression.^90^ Additionally, Parkin can promote the degradation of TRAF2 and TRAF6, thereby inhibiting the nuclear factor-kappa-B (NF-κB) signaling pathway and inducing tumor apoptosis.^91^

### Nonclassical ubiquitination

Although the UPS is exclusive to eukaryotes, a recent report revealed that the SidE (siderophore E) effector family could perform atypical ubiquitination on a variety of host proteins.^92^ It is derived from *Legionella pneumophila* and works as an E3 ligase independent of E1 and E2 enzymes. The diverse strategies adopted by SidE are divided into two steps. First, the mART domain of SidE catalyzes the attachment of ADP-ribose to Arg_42_ of Ub and forms ADP-ribosylated Ub (ADPr-Ub). Second, the PDE domain of SidE further cleaves the phosphodiester bond in ADPr-Ub to form phospho-ribosylated Ub (Pr-Ub), which is covalently attached to the Ser of the substrate through the PDE domain of SidE. Further mechanistic studies successfully revealed a high-resolution crystal structure of the pre-reaction (SidE protein alone), the first step reaction complex (mART-Ub-NAD) and the second step reaction complex (PDE-Ub-ADP ribose). Combined with a large number of biochemical experiments and mutant analysis, the interaction between the novel E3 ligase SidE with Ub and ligand is completely presented.^93,94^ These exciting findings not only open a new chapter in the ubiquitination field but also provide a theoretical basis for developing targeted drugs.

### Ub-like proteins

In addition to Ub, the Ub superfamily also contains Ub-like (UBL) proteins, which includes NEDD8, SUMO, FAT10, ISG15, ATG8, ATG1, HUB1 and FUB1. These UBL proteins not only have sequence homology and structural similarity to Ub but also use a similar enzymatic cascade to modify their substrate proteins.^95,96^ Due to space limitations, we will mainly discuss neddylation and SUMOylation below in this review.

#### NEDD8

In the UBL superfamily, NEDD8 has the highest homology with Ub and is indispensable in various biological processes. The specific attachment of NEDD8 to the substrate protein is called neddylation, which is a dynamic and reversible process. To date, there are many kinds of NEDD8-specific E3 ligases that determine the specificity of substrates, along with one E1 (NEDD8 activating enzyme, NAE) and two NEDD8-specific E2 ligases. The NEDD8 modification can be reversed by the COP9 signalosome (CSN), which deconjugates NEDD8 from the cullin protein.^96–98^

Unlike ubiquitination, neddylation does not degrade the substrate. However, as a PTM, neddylation also regulates the activation of substrates and subsequently controls a variety of cellular biological functions, such as cell cycle regulation and signal transduction. For example, neddylation mediates the biological function of the cullin-RING ubiquitin ligase (CRL) family and regulates the activity of the E3 complex by cullins, the key subunit of CRLs. Blocking the neddylation of cullins leads to substrate accumulation.^99,100^

#### Small ubiquitin-related modifier

Small ubiquitin-related modifier (SUMO), a widely expressed UBL protein in eukaryotes, is named for its similar structure and enzymatic cascade with Ub.^101^ SUMOylation is the process in which SUMO links to a substrate by forming an isopeptide bond between its terminal glycine and the lysine of the substrate.^96,102^ Currently, more than 500 substrates have been reported to undergo SUMOylation and take part in regulating the localization, stability and activity of many proteins.^103,104^ For instance, the SUMOylation of RPA1 (RPA subunit) regulates the affinity between RPA and RAD51 and promotes homologous recombination (HR)-mediated DNA double-strand break (DSB) repair.^105^ RNF4, a SUMO-targeted E3 ligase, has been identified as the link between ubiquitination and SUMOylation. SUMOylation of PML recruits RNF4 and triggers its degradation in a ubiquitination-proteasome-dependent way.^106–108^ Therefore, SUMOylation takes part in a variety of cellular physiological activities, such as gene stability maintenance and transcriptional regulation, and aberrant SUMOylation is closely related to the development and progression of certain diseases, including cancer.

### Deubiquitinating enzymes

Ubiquitination, a dynamic and reversible process, is regulated by DUBs and E3 ligases.^109^ DUBs belong to the family of Cys proteases and cleave the isopeptide bond (the attachment of Ub to lysine) or the peptide bond (the connection of Ub to the N-terminal methionine of the protein) with high specificity.^70,109^

Currently, the human genome encodes no less than 100 DUBs (Fig. 1a). According to their sequence and structural similarities, they can be divided into six families: ubiquitin-specific proteases (USPs), ubiquitin carboxyl-terminal hydrolases (UCHs), otubain proteases (OTUs), Machado-Joseph disease protein domain proteases (MJDs), JAMM/MPN domain-associated metallopeptidases (JAMMs) and monocyte chemotactic protein-induced proteins (MCPIPs). Among them, all DUBs are Cys proteases except the JAMM family of metalloproteinases. These enzymes are capable of directly binding to different types, topologies or lengths of Ub chains and removing Ub chains from the substrate.^110^ Engineered deubiquitination synthesis reveals that the OTU specifically removes the K29 linkage Ub chain from the substrate,^111^ and the JAMM, such as AMSH, AMSH-LP, BRCC36 and POH1, are often specific for the Ub chain for K63 linkage ubiquitination.^112,113^ CYLD is more likely to act on linear ubiquitination and the K63 linkage Ub chain.^114^ Similarly, OTU domain-containing ubiquitin aldehyde-binding protein 1 (OTUB1) specifically acts on K48-linked ubiquitination,^115,116^ with Cezanne specifically removing the K11 linkage Ub chain,^117,118^ and TRABID specifically recognizing the K29-linked or K33-linked Ub chain.^119^ These specific Ub-type deubiquitinating enzymes cannot remove the last molecule of Ub-modified on the substrate, which may generate a monoubiquitinated substrate protein.

To date, many DUBs have been found to be associated with p53 regulation in tumorigenesis. For example, USP7 regulates the stability of both p53 and Mdm2 and maintains p53 ubiquitination levels;^120^ USP2 mediates the stability of Mdm2;^121^ USP10 modulates p53 localization and stability;^122^ OTUB1 abrogates p53 ubiquitination and activates p53.^123^ Interestingly, USP10 can stabilize both mutated and wild-type p53, with a dual role in tumorigenesis. USP11 participates in the regulation of DNA DSB repair. USP11 is often overexpressed in cancer and induces resistance to poly(ADP-ribose) polymerase 1 (PARP1) inhibitors.^124^

## Ubiquitination in tumor metabolism regulation

### Ubiquitination in the mTORC1 signaling pathway

As an important nutrient and key environmental stimulus, amino acids play a critical role in the mechanistic target of rapamycin complex 1 (mTORC1) signaling pathway. The mechanism of the amino acid-induced mTORC1 signaling pathway is still under continuous research. One well-demonstrated model has proposed that the activation of mTORC1 is induced by amino acid sensing cascades, including Rag GTPase, Ragulator and vacuolar H+-ATPase (v-ATPase), at lysosomes. During this process, amino acids can promote RagA/B binding to GTP, which is essential for mTORC1 lysosome localization.^125–127^ Moreover, many regulators of RagA/B have been identified, and these include the SLC38A9 functioning as the guanosine exchange factor (GEF) of RagA/B,^128^ Sestrin2 identified as the guanine nucleotide dissociation inhibitor (GDI) of RagA/B,^129^ and the GATOR1 complex acting as a GTPase-activating protein (GAP) to RagA/B.^130^ However, the role of ubiquitination in the RagA-mTORC1 pathway in response to amino acids is still poorly understood.

#### Ubiquitination of RagA

**Fig. 2:**
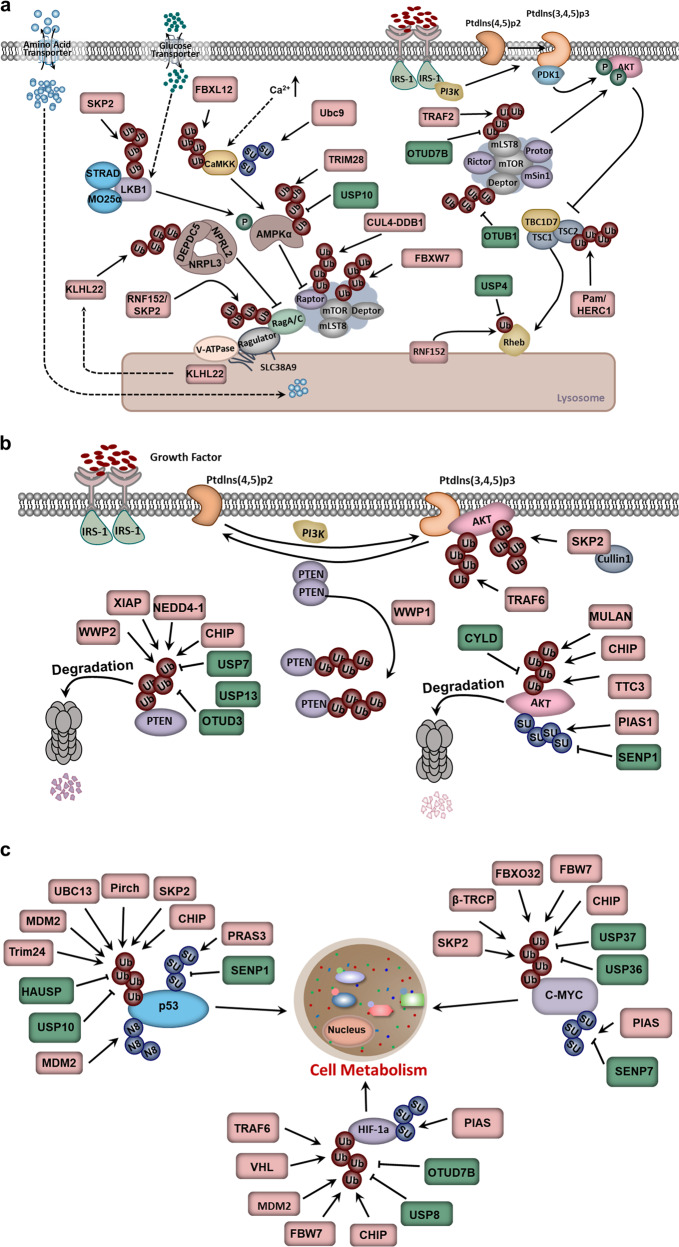
Ubiquitination in tumor metabolism regulation. **a** Ubiquitination in the mTORC1 signaling pathway. **b** Ubiquitination in the PTEN-AKT signaling pathway. **c** Ubiquitination of key transcription factors in cell metabolism regulation

Recently, RagA and mTORC1 were found to be inactivated upon acute amino acid withdrawal. In this study, RagA was modified by polyubiquitination in an amino acid-sensitive manner. By screening a series of E3 ligases, RNF152, a lysosomal E3 ligase, was identified to mediate K63-linked polyubiquitination of RagA. In addition, ubiquitination of RagA recruited GATOR1, led to the inactivation of RagA and caused mTORC1 release from the lysosomal surface, thereby blocking the inactivation of the mTORC1 signaling pathway.^61^ Moreover, SKP2, another E3 ligase, could mediate RagA polyubiquitination on lysine 15.^131^ Thus, the polyubiquitination of RagA plays an important role in regulating the mTORC1 signaling pathway (Fig. 2a).

#### Ubiquitination of mTOR

Undoubtedly, mTOR occupies a decisive position in the amino acid-induced mTORC1 signaling pathway. As mentioned above, being located on lysosomes via RagA deubiquitination is the premise of mTORC1 activation.^130,132–134^ In addition to RNF152/SKP2, TRAF6, an E3 ligase, is also reported to regulate mTOR translocation to the lysosome in response to amino acid stimulation by catalyzing the K63 ubiquitination of mTOR in the form of the p62-TRAF6 heterodimer complex. Thus, TRAF6 regulates autophagy and cancer cell proliferation by activating mTORC1.^135^ In addition to K63 ubiquitination, other types of polyubiquitin linkages have also been identified on mTOR. K48 ubiquitination is reported to be involved in the stability of mTOR. In this process, FBXW7 directly binds to mTOR and mediates its degradation by the proteasome (Fig. 2a).^136^ These results highlight the dominant role of ubiquitination in the mTORC1 pathway and reveal that different types of ubiquitination linkages lead to different functions.

#### Ubiquitination of DEPDC5

Amino acid stimulation can abolish the interaction between Sestrin2/CASTOR1/2 and the GATOR2 complex, which is essential for the activation of Rag GTPase. GATOR2 dissociates from Sestrin2/CASTOR1/2 and activates RagA/B by inhibiting the activity of GATOR1, which consists of DEPDC5, NPRL3 and NPRL2, and displays GAP activity to RagA/B.^129,130,132,137^ In addition, ubiquitination is also involved in the regulation of GATOR1 activity, in which Cullin3-KLHL22 E3 ligase promotes K48 linkage polyubiquitination of DEPDC5 and mediates its degradation by proteasomes under amino acid-stimulated conditions (Fig. 2a). KLHL22 plays a conserved role in the mTORC1-mediated autophagy, cell size and regulation of the nematode lifespan through DEPDC5. Moreover, the expression of KLHL22 is significantly negatively correlated with DEPDC5 in patients with breast cancer. Therefore, pharmacological interventions targeting KLHL22 may have therapeutic potential for relevant diseases, such as breast cancer and age-related diseases.^138^

#### Ubiquitination of mLST8

mTOR predominantly exists in two multicomponent kinase complexes, mTORC1 and mTORC2, which are structurally related but functionally distinct. The mTORC1 and mTORC2 signaling pathways are not independent.^127^ The activation of mTORC1 is inseparable from AKT activated by mTORC2, and the feedback inhibition of mTORC2 activation requires mTORC1-mediated Sin1 phosphorylation.^139^ mTORC2 contains six components, of which mTOR, DEPTOR and mLST8 are identical to mTORC1. Therefore, the dynamic assembly of mammalian lethality with SEC13 protein 8 (mLST8) in the two complexes is important for both complexes. Previous studies have shown that the K63 linkage polyubiquitination of mLST8, promoted by TRAF2, determines the homeostasis of mTORC1 formation and activation. Specifically, the K63 linkage polyubiquitination of mLST8 disrupts its interaction with the mTORC2 component Sin1 to favor mTORC1 formation. In addition, the deubiquitinating enzyme OTUD7B was reported to facilitate the formation of mTORC2 by removing the polyubiquitin chain on mLST8 and then promoting the interaction between mLST8 and Sin1. Collectively, the dynamic assembly and activation of mTORC1 and mTORC2 are dependent on the ubiquitination of mLST8, further demonstrating the importance of ubiquitination in the mTOR signaling pathway (Fig. 2a).^140^

#### Ubiquitination of DEPTOR

DEP domain-containing mTOR-interacting protein (DEPTOR) is an important component and negative regulator of both mTORC1 and mTORC2.^141^ Its stability is governed in a Ub-proteasome pattern by the E3 ligase beta-transducin repeat containing protein 1 (β-TrCP1), simultaneously proven by three different teams.^142–144^ In these studies, DEPTOR was recognized by β-TrCP1 via its degron sequence and subsequently ubiquitinated and degraded. Moreover, DEPTOR accumulation upon β-TrCP1 knockdown or the degron mutation could promote autophagy by inactivating mTORC1 (Fig. 2a).

The regulatory mechanisms of DEPTOR stability have also been explored. OTUB1 specifically interacts with DEPTOR via its N-terminal domain, removes the Ub chain on DEPTOR and stabilizes DEPTOR via DUB activity in an Asp88-dependent but not Cys91-dependent manner (Fig. 2a). Thus, β-TrCP1 and OTUB1 can balance cell survival and autophagy by activating mTORC1 through regulating DEPTOR ubiquitination, which also illuminates the importance of ubiquitination in the mTORC1 signaling pathway.^145^

#### Ubiquitination of TSC-Rheb

As a major regulator of Ras homolog enriched in brain (Rheb), the TSC complex is the central node for many growth and stress signals, ranging from growth factors, glucose, oxygen and energy to oncogenes and tumor suppressors. TSC2, a short-lived protein, is regulated by PTM in response to upstream signals.^146^ ERK- and AKT-mediated phosphorylation of TSC2 can result in the activation of Rheb,^147,148^ while ubiquitination can regulate the stability of TSC2. For example, TSC2 can bind to FBW5, a compound of the FBW5-DDB1-CUL4-ROC1 E3 ligase. The overexpression of FBW5 or CUL4A promotes TSC2 ubiquitination and degradation. Thus, FBW5 is a specific E3 ligase targeting TSC2 for its degradation and promoting TSC complex turnover (Fig. 2a).^149^

mTORC1 is recruited to lysosomes, where it is activated by its interaction with GTP-bound Rheb.^133^ The ubiquitination of Rheb regulates its activity. It has been reported that the ubiquitination of Rheb governs its nucleotide-bound status and controls the transformation between Rheb-GDP and Rheb-GTP. The lysosomal E3 ligase RNF152 can induce Rheb ubiquitination and promote its binding to the TSC complex in an epidermal growth factor (EGF)-sensitive manner. Upon growth factor stimulation, USP4 removes the Ub chain from Rheb in an AKT-dependent manner, which leads to the release of Rheb from the TSC complex, resulting in the subsequent activation of both Rheb and mTORC1. Therefore, the ubiquitination of Rheb, determined by RNF152 and USP4, also plays an important role in mTORC1 activation and consequent tumorigenesis (Fig. 2a).^150^

### Ubiquitination in the adenylate-activated protein kinase signaling pathway

Adenylate-activated protein kinase (AMPK), the master sensor of energy in cells and organisms, is the core in regulating intracellular metabolic homeostasis, and its mutation is associated with tumorigenesis.^151,152^ When the cellular level of ATP decreases and AMP/ATP increases, the activation of AMPK increases. For instance, in response to a high AMP/ATP ratio in the cytosol, AMPK enhances glucose uptake and utilization by regulating key proteins in the cellular metabolic pathway, as well as fatty acid oxidation, to produce more energy.^151,153^ In addition to being phosphorylated, AMPK can also undergo ubiquitination. Specifically, the E3 ligase MAGE-A3/6-TRIM28 can ubiquitinate AMPK and promote its degradation. Furthermore, two homologs, MAGE-A3 and MAGE-A6, originally expressed only in the male germline, are reactivated in tumors. In mice, overexpressing MAGE-A3/A6 in cell lines promotes tumor growth and metastasis. In this process, MAGE interacts with the E3 ligase TRIM28, which controls the stability of AMPKα by mediating the K48 linkage polyubiquitination of AMPKα (Fig. 2a).^154^

In addition to K48 linkage polyubiquitination, the activation of AMPKα is also regulated by K63-specific polyubiquitination, which may mask its structure to block the access of liver kinase B1 (LKB1) and inhibit the activation of AMPKα. The deubiquitinating enzyme USP10 can remove the Ub chain from AMPKα and promote AMPKα activation by facilitating LKB1-mediated AMPKα phosphorylation, thereby participating in glucose and lipid metabolism in cells.^155^ In addition, ubiquitination also acts on LKB1. Generally, LKB1 functions as an oncoprotein and is activated by a complex with STRAD and MO25.^156–158^ SKP2 promotes the K63 polyubiquitination of LKB1 and plays an important role in LKB1 activation by maintaining the intact LKB1-STRAD-MO25 complex (Fig. 2a). Additionally, in a hepatocellular carcinoma model, SKP2-mediated LKB1 polyubiquitination is required for its activation and cell survival.^159^

In addition, ubiquitination is also involved in the regulation of the calcium/calmodulin-dependent protein kinase kinase 2 (CaMKK2)–AMPK signaling pathway. For example, the stability of CaMKK2 is controlled by the E3 ligase Fbxl12, which facilitates the degradation of CaMKK2 by promoting its ubiquitination (Fig. 2a).^160^ Thus, to maintain intracellular metabolic homeostasis, ubiquitination should not be ignored in the regulation of AMPK.

### Ubiquitination in the PTEN-AKT signaling pathway

Unlike amino acid stimulation, growth factors are sensed by PTEN-AKT. It has been validated that both the PTEN-AKT and mTOR signaling pathways are important for the growth factor response. However, the two pathways were not unified until the identification of two key proteins: the small GTPase Rheb and its negative regulator TSC complex (Fig. 2a).^148,161–163^

#### Ubiquitination of PTEN

PTEN, a tumor suppressor and lipid phosphatase, plays an important role in tumorigenesis by inhibiting the PI3K signaling pathway. Generally, PTEN can be ubiquitinated and deubiquitinated. Nedd4-1, WW domain-containing ubiquitin E3 ligase 2 (WWP2), X-linked inhibitor of apoptosis protein (XIAP) and C-terminus of HSC70-interacting protein (CHIP) have been identified as the specific E3 ligases for PTEN, and the ubiquitination of PTEN mediated by each of them has different functions.^164–167^ For example, Nedd4-1, an E3 ligase of the HECT family, can promote both monoubiquitination and polyubiquitination of PTEN at K13 and K289, leading to the cytoplasmic localization and subsequent degradation of PTEN. PTEN is usually stable and not polyubiquitinated in the nucleus. The monoubiquitination of PTEN induces its translocation from the nucleus to the cytoplasm, and further polyubiquitination functions as a proteolytic signal to degrade PTEN via the proteasome.^168^ Moreover, WWP2 is also found to ubiquitinate PTEN and regulate cell apoptosis by mediating PTEN degradation.^165^ Additionally, the E3 ligase WWP1-induced PTEN ubiquitination inhibits PTEN dimerization, membrane recruitment and function. Inhibiting the activity of WWP1 leads to PTEN reactivation and blocks MYC-driven tumorigenesis.^169^ Moreover, the E3 ligases XIAP and CHIP can also target PTEN for ubiquitination and degradation and further activate the AKT signaling pathway (Fig. 2b).^166,167^

Due to the reversibility of ubiquitination, the deubiquitination of PTEN has also attracted the attention of researchers. USP7, a highly expressed DUB in prostate cancer and progressive multifocal leukoencephalopathy (PML), plays a direct role in PTEN deubiquitination and regulates its localization rather than protein stability.^170^ In addition, USP13 and OTUD3 can interact with PTEN and remove its polyubiquitin chain. Subsequently, blocking the degradation of PTEN inhibits the activity of the AKT signaling pathway and tumor growth (Fig. 2b).^171,172^

#### Ubiquitination of AKT

As a critical upstream target of the mTORC1 signaling pathway, AKT kinase transfers growth factor signals from the extracellular environment to the intercellular space. Its activation, which depends on the localization to the plasma membrane and is associated with K63-linked ubiquitination, is essential for cell growth, proliferation and metabolism.^173,174^ TRAF6, SKP2, tetratricopeptide repeat domain 3 (TTC3), CHIP, Nedd4 and MULAN have been identified as E3 ligases for AKT and participate in AKT kinase activation. TRAF6 is a direct E3 ligase for AKT in response to IGF-1 stimulation, and K63 polyubiquitination by TRAF6 is necessary for AKT membrane recruitment, phosphorylation and activation. The cancer-associated AKT mutation displays an increasing trend in AKT ubiquitination.^175^ In addition, SKP2 is also the E3 ligase for ErbB-receptor-mediated AKT ubiquitination. In a breast cancer metastasis model, SKP2 deficiency decreases the activation of the AKT kinase.^176^ Moreover, K48 linkage ubiquitination was also identified to regulate the stability of AKT instead of its activation. In addition, many studies have been identified that CHIP, MULAN and TTC3, an E3 Ub ligase, can ubiquitinate AKT and mediate its degradation (Fig. 2b).^177–180^

Corresponding to ubiquitination, “eraser” DUBs can also regulate protein degradation, localization, activation and protein–protein interactions of AKT. The cylindromatosis (CYLD), a well-known tumor suppressor, can interact directly with AKT and deubiquitinate its K63-linked ubiquitination in response to the stimulation of growth factors, which results in K48 linkage polyubiquitination via BRCA1 or TTC3 (Fig. 2b).^181^ The loss of CYLD accelerates tumorigenesis and triggers cisplatin resistance in melanoma and oral squamous cell carcinoma.^182,183^ Thus, CYLD is considered a molecular switch for the ubiquitination of AKT and determines the localization and activation of AKT during cancer progression.

To date, it has been well documented that the AKT kinase can be modified by phosphorylation, ubiquitination, acetylation, methylation and hydroxylation.^184,185^ Moreover, SUMOylation can also be responsible for AKT activation (Fig. 2b). Lysine 276, located in the SUMOylation consensus motif, is essential for AKT activation, while the mutation of K276R can reduce the SUMOylation of AKT, and AKT E17K can mediate cell proliferation, migration and tumorigenesis.^186^

### Ubiquitination of key transcription factors in cell metabolism regulation

Transcription factors also play crucial roles in regulating cellular metabolism. When cells are in a state of limited energy intake or starvation, transcription factors can activate the related genes in glycolysis and the tricarboxylic acid cycle, increase hepatic glucose production, reduce insulin secretion and provide a substrate for gluconeogenesis. Among them, hypoxia-inducible factor-1α (HIF-1α), Myc and p53 are closely related to cell metabolism.^151,187,188^

#### Ubiquitination regulates HIF-1α

HIF1, a transcription factor widely expressed under hypoxic conditions, is the key regulator of oxygen homeostasis in cells.^189^ It can induce the expression of many glycolytic genes, such as glucose transporter member 1, hexokinase 1 and hexokinase 2, lactate dehydrogenase A, monocarboxylate transporters 4 and PDK1, which are indispensable in glucose uptake. HIFs include three subtypes: HIF1, HIF2 and HIF3. They are composed of α and β subunits, wherein the α subtype, which is sensitive to oxygen, is easily degraded via the proteasome pathway; and in contrast, the β subunit is more stable.^190,191^

Due to the important role of HIF in cells, many studies have been performed to investigate the regulatory mechanism of HIF.^192,193^ Among them, E3 ligases and DUBs have been found to regulate the stability of HIF. Under normal conditions, HIF-1α is extremely unstable. The tumor suppressor E3 ligase von Hippel-Lindau (VHL), which is widely involved in tumor vascularization, interacts with HIF-1α in a proline hydroxylation-dependent manner and mediates its degradation, thereby inhibiting tumor growth (Fig. 2c).^194^

It has been found that glycogen synthase kinase 3 (GSK3β) phosphorylates HIF-1α and promotes K48 polyubiquitination by FBW7, thereby mediating the degradation of HIF-1α and inhibiting angiogenesis, cell migration and tumor growth.^195^ On the other hand, the FBW7-mediated proteolytic signal can be removed by the deubiquitinating enzyme USP28.^196^ In addition to VHL, the tumor suppressors p53, Tap73 and PTEN also recruit the E3 ligase Mdm2 to HIF-1α, leading to the ubiquitination and degradation of HIF-1α by the proteasome.^197^ Unlike K48 linkage polyubiquitination, TRAF6 can mediate the K63 linkage polyubiquitination of HIF-1α and block its degradation (Fig. 2c).^198^ Moreover, the E3 ligase FBXO11 can reduce the mRNA level of HIF-1α but has no effect on its protein stability.^199^

Similarly, HIF-1α can also be deubiquitinated. For example, OTU deubiquitinase 7B (OTUD7B) can deubiquitinate HIF-1α and inhibit its degradation by the lysosome.^200^ By screening an siRNA library, the deubiquitinating enzyme USP8 interacts with HIF-1α, removes the Ub chain from HIF-1α, and maintains its expression and transcriptional activity under normal oxygen.^201^ Moreover, HIF-1α can also undergo SUMOylation. Overexpressing the SUMO molecule and SUMO ligase in lymphatic endothelial cells can induce the SUMOylation of HIF-1α and maintain the stability and transcriptional activity of HIF-1α (Fig. 2c).^202^

#### Ubiquitination regulates c-Myc

The transcription factor c-Myc can regulate cell proliferation, metabolism and metastasis by mediating a variety of cellular metabolism pathways, such as glucose metabolism, fatty acid and nucleotide biosynthesis. c-Myc is involved in glucose uptake and glycolysis, and its activation upregulates the expression of glucose transporters and hexokinases.^203,204^ As a very unstable protein with a very short half-life, c-Myc can be degraded in a proteasome-dependent manner, and many studies have identified the E3 ligase and DUB of c-Myc. For example, in the G1 to S phases of the cell cycle, the E3 ligase SKP2 can interact with c-Myc and mediate its degradation by ubiquitination, thereby blocking the cell cycle and inhibiting tumorigenesis.^205,206^ Additionally, the phosphorylation of c-Myc on Thr58 by GSK3 promotes its interaction with Fbw7 and facilitates K48 linkage polyubiquitination. The subsequent degradation inhibits cell proliferation and tumor growth. Missense mutations of Fbw7 are found in many malignancies; for example, the mutation R465C fails to degrade c-Myc in T-cell acute lymphoblastic leukemia.^207^ In addition to Fbw7, other E3 ligases, such as β-TrCP1, CHIP and FBXO32, can also ubiquitinate c-Myc, mediate its subsequent degradation and inhibit tumorigenesis.^208–210^ Moreover, the USP37 and USP36 can promote tumorigenesis by stabilizing c-Myc.^211,212^ By mass spectrometry, SUMO ligase protein inhibitor of activated STAT (PIAS) and Sentrin-specific protease 7 (SENP7) were also found to control the SUMOylation of c-Myc at K326 and regulate its ubiquitination and degradation (Fig. 2c).^213^

#### Ubiquitination regulates p53

p53, one of the most important tumor suppressors, works in multiple cellular processes, such as cell cycle regulation, DNA repair and apoptosis. In addition, p53 also plays an important role in cell metabolism by inhibiting glycolysis and promoting oxidative phosphorylation in response to nutrient stimulation.^214,215^

Under the stimulation of low carcinogenicity and genotoxicity, p53 is persistently expressed, but its protein level is often maintained at low levels. Under the stimulation of the external environment, the degradation of p53 is inhibited, resulting in the improvement of its stabilization and transcriptional activity.^214^ To date, more than 15 E3 ligases of p53 have been identified, and they are divided into the RING family (Mdm2, Pirh2, Trim24, Cul1/Skp2, Cul4a/DDB1/Roc, Cul5, Cul7, Synoviolin, Cop1, CARP1/2, CHIP, UBE4B) and HECT family (ARF-BP1, Msl2/WP1) ligases. Moreover, both K48- and K63-linked ubiquitination have been found on p53. The former can promote the degradation of p53 via the proteasome, while the latter is required for the translocation of p53 to the cytoplasm. More specifically, the E3 ligases Mdm2, Pirh2, Trim24, Cul1/Skp2, Cul4a/DDB1/Roc, Cul5, Synoviolin, Cop1, CARP1/2, ARF-BP1, Msl2/WP1, CHIP and UBE4B mediate K48 linkage polyubiquitination, which degrades p53 via the proteasome, while Cul7-mediated polyubiquitination regulates the localization and activity of p53 (Fig. 2c).^9,216,217^

As mentioned above, deubiquitination is also a key regulatory step in cell metabolism. The deubiquitination enzymes HAUSP and USP10 can remove the proteolytic signal of p53 and abolish its degradation. HAUSP, which is localized in the nucleus, can prevent the degradation of p53 by deubiquitinating even under the circumstance of highly expressed Mdm2.^120,218^ Unlike HAUSP, USP10 is generally located in the cytoplasm. In the case of DNA damage, the phosphorylation of USP10 at Thr42 and Ser337 mediated by ATM is essential for its translocation to the nucleus, where USP10 induces the deubiquitination of p53 and makes p53 work as a tumor suppressor (Fig. 2c).^122^

In addition to ubiquitination, p53 can also undergo other ubiquitination-like modifications, such as neddylation and SUMOylation. For example, Mdm2 can inhibit p53-mediated transcriptional activity via neddylation on K370, K372 and K373 of p53.^219^ SUMOylation of p53 can regulate the transcriptional activity under genotoxic stress (Fig. 2c).^220^

## Ubiquitination in immunological TME modulation

The innate immune system can recognize invading pathogenic microorganisms by inducing the expression of proinflammatory and anti-infective genes. During the process of tumorigenesis, premalignant lesions, regarded as invaders, can lead to inflammation and activate local innate immune surveillance to the malignant cells in the early stages.^221^ Then, the inflammatory, immunological and metabolic processes of the tumor and the tumor-draining lymph nodes (TDLNs), constituting the immunological TME, are also reprogrammed.^222^ According to Dvorak’s 1986 comment, malignancies are regarded as “wounds that do not heal”.^223,224^ As an important risk factor for malignancy, chronic immune activation and inflammation persistently promote TME formation by providing inflammatory mediators such as TNF-α, IL-1β, IL-6 and TGF-β and ultimately lead to angiogenesis and antitumor immunity.^225,226^

Ubiquitination, a ubiquitous PTM in cells, appears to be a critical mediator of the host cell defense and immunological TME modulation by regulating cell signal transduction pathways. On the one hand, as a multifunctional signal regulator, ubiquitination can precisely regulate the process of the immune response in a time and space manner.^227^ On the other hand, it can effectively induce antitumor immunity by mediating the degradation of key signal transduction molecules to stabilize and maintain the balance between tumor suppressors and oncoproteins.^228,229^ The Toll-like receptor (TLR), RIG-like receptor (RLR) and DNA recognition receptor signaling pathways are very important in the immune system; thus, we introduce the functions of ubiquitination in TLR, RLR and DNA recognition receptor signaling pathways, and related molecular regulatory mechanisms are relatively highly studied.

### Ubiquitination in the TLR signaling pathway

**Fig. 3:**
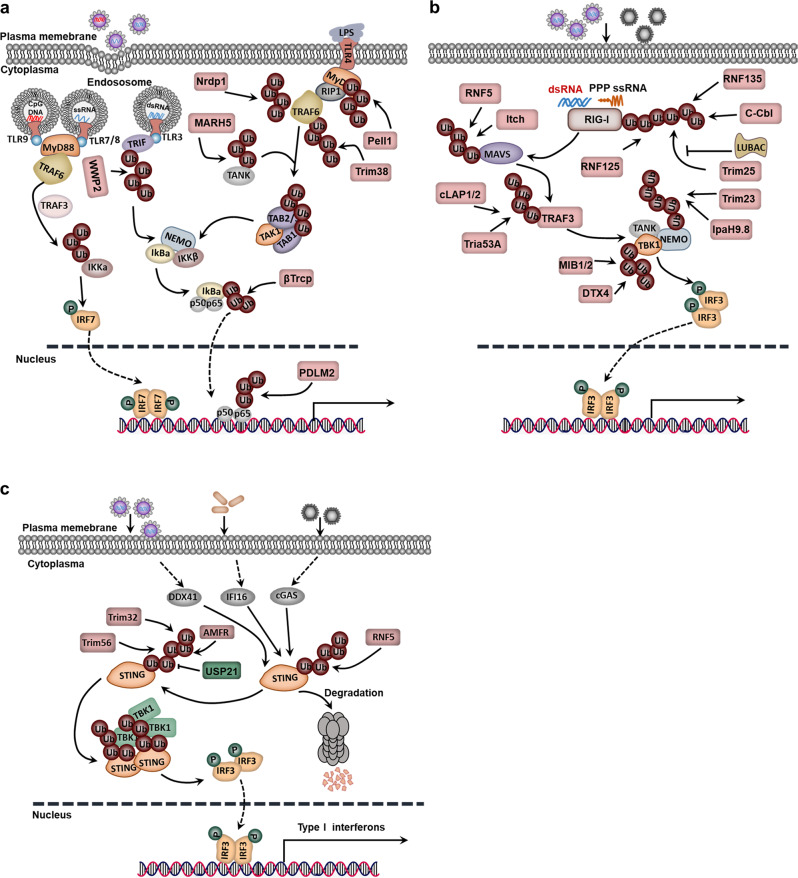
Ubiquitination in immunological tumor microenvironment (TME) modulation. **a** Ubiquitination in the TLR signaling pathway. **b** Ubiquitination in the RLR signaling pathway. **c** Ubiquitination in the STING-dependent signaling pathway

As innate immune receptors, TLRs are involved in the recognition of microorganisms by the immune system. Generally, TLRs recognize a conserved component of the pathogen and then activate the signaling pathway.^230^ TLR signaling in immune and inflammatory cells of the TME also induces the production of proinflammatory cytokines and leads to the polarization of tumor-associated macrophages (TAMs), activation of protumorigenic functions of immature myeloid cells and transformation from fibroblasts into cancer-associated fibroblasts (CAFs).^231^ TLR, a family of receptors, has 13 members. Among them, TLR4/7/8/9 activates the MyD88-dependent signaling pathway and subsequently elevates the activity of the downstream TRAF6. In the TLR4 signaling pathway, the K63 polyubiquitin chain catalyzed by TRAF6 recruits the TGF-β-activated kinase 1 (TAK1) complex and IκB kinase (IKK) complex and then increases the expression of inflammatory factors downstream of NF-κB. The K63 polyubiquitin chain also recruits TRAF3, IKKα and IRF7 and ultimately increases the expression of type I interferon in the TLR7/8/9 signaling pathway.^232,233^ In addition, the K63 polyubiquitination of receptor-interacting serine/threonine-protein kinase 1 (RIPK1), catalyzed by the E3 ubiquitin ligase Peli1, plays an important role in the TIR-domain-containing adapter-inducing interferon-β (TRIF)-dependent TLR signaling pathway, which significantly enhances the activation of NF-κB by transferring NF-κB or IRF3 to the nucleus to regulate the transcription of target genes.^234^ MARCH5, an E3 ligase located on mitochondria, catalyzes K63-linked polyubiquitination of TRAF family member-associated NF-κB activator (TANK) and then enhances the activation of the TLR signaling pathway (Fig. 3a).^235^

NF-κB signaling pathway inhibitors are degraded by the ubiquitination-proteasome pathway. For example, the transcription factor NF-κB is retained in the cytoplasm due to its interaction with the inhibitor IκBα in the remaining cells, while lipopolysaccharide (LPS) stimulation induces the phosphorylation of IκBα by IKKβ and degradation by the E3 ligase β-TrCP1.^236^ In some cases, polyubiquitination at K48 and K63 can also synergistically promote the activation of signaling pathways. For example, MyD88 is crucial for gathering TRAF6, TRAF3 and cIAP1/2 in the TLR4-MyD88 signaling pathway. More specifically, TRAF6, recruited by MyD88, activates cIAP by catalyzing the K63-linked polyubiquitination of cIAP, while activated cIAP induces the K48 polyubiquitination of TRAF3, leading to the degradation of TRAF3 by the proteasome.^237^ TRAF6 can also result in the ubiquitination of ECSIT and increase mitochondrial and cellular TLR-induced ROS generation.^238^ Recently, USP4 has been identified as a new DUB of TRAF6 and can negatively regulate the NF-κB signaling pathway.^239^

The ubiquitination process is also a safeguard to prevent tumorigenesis by inhibiting the overactivation of NF-κB at multiple sites. A negative regulator A20 can cooperate with RNF11, ITCH and TAX1BP1 to remove the K63-linked polyubiquitin chain catalyzed by TRAF6 from cIAP.^240^ Additionally, Nrdp1, Trim38, WWP2 and PDLIM2 trigger the K48-linked polyubiquitination of MyD88, TRAF6, TRIF and p65, respectively, and promote the degradation of target proteins by proteasomes (Fig. 3a).^241–244^

### Ubiquitination in the RLR signaling pathway

The RLR family comprises three members: retinoic acid-inducible gene 1 (RIG-I), melanoma differentiation-associated protein 5 (MDA5) and laboratory of genetics and physiology 2 (LGP2). The RIG-I/MDA5 receptor recognizes and binds to viral RNA and regulates the expression of antiviral genes through the MAVS-TBK1-IRF3 signaling pathway.^245^ Many E3 ligases are involved in regulating the downstream signaling of MAVS. For example, the E3 ligase Trim25 catalyzes the K63 polyubiquitination of RIG-I. Subsequently, MAVS is recruited, and the activation signal is transferred to the MAVS signal complex.^246^ Another is LUBAC, which decreases the activation of RIG-I by inhibiting the binding of Trim25 and RIG-I or mediating the polyubiquitination and degradation of Trim25.^247^ Similar to Trim25, RNF135 (also known as Riplet) also catalyzes the K63 polyubiquitination of RIG-I and activates the RIG-I signaling pathway.^248^ Additionally, the E3 ligase MIB1/2 regulates the activation of TANK-binding kinase 1 (TBK1) by catalyzing TBK1 K63-linked polyubiquitination. The K27-linked polyubiquitination of NF-κB essential modulator (NEMO), which is mediated by Trim23 and Shigella effector IpaH9.8, can also promote the activation of the TBK1 and IKK complexes (Fig. 3b).^249,250^ Thus, E3 ligases have a key regulatory function in the RLR signaling pathway, and the regulation of ubiquitination on the immune response is complex and precise.

To avoid tumorigenesis caused by excessive activation of the RLR signaling pathway, host cells inhibit the overproduction of downstream inflammatory factors and interferons by ubiquitinating and degrading key proteins in the RLR signaling pathway. The E3 ligase RNF125 catalyzes the K48 linkage ubiquitination of RIG-I/MDA5 and promotes the degradation of RIG-I/MDA5 through the proteasome.^251^ More importantly, upon stimulation with an RNA virus, the lectin family member Siglec-G recruits the E3 ligase c-Cbl, catalyzes the K48-linked ubiquitination of RIG-I, and promotes the degradation of RIG-I.^252^ As a pivotal protein of the RLR signaling pathway, mitochondrial antiviral signaling (MAVS) is also regulated by ubiquitination. The poly(rC)-binding protein PCBP2 recruits the E3 ligase ITCH, which catalyzes the K48 ubiquitination of MAVS, regulates its degradation, and inhibits the activation of the RLR signaling pathway mediated by MAVS.^253^ Many E3s have been identified to regulate the stability of MAVS downstream signaling components. For example, NACHT, LRR and PYD domains-containing protein 4 (NLRP4) recruits the E3 ligase DTX4 and promotes the ubiquitination and degradation of TBK1.^254^ Triad3A catalyzes the K48 polyubiquitination of TRAF3.^255^ The E3 ligase RNF5 promotes the K48-linked ubiquitination and degradation of MAVS (Fig. 3b).^256^

### Ubiquitination in the STING-dependent signaling pathway

STING, an adapter transmembrane protein residing in the endoplasmic reticulum (ER), is an important innate immune sensor for tumor detection.^257–259^ The STING pathway is activated by antigen-presenting cells (APCs) and produces type I IFNs. Subsequently, adequately activated APCs in the TME induce CD8^+^ T cell priming and lead to adaptive anticancer immune responses.^260^ Recently, many DNA-binding proteins have been reported in the cytoplasm and include cGAS, Mre11, IFI16 (p204), DDX41 and DNA-PKcs. They recognize DNA in the cytoplasm and strongly initiate the type I interferon gene through the STING-TBK1-IRF3 signaling axis. In response to the stimulation of cytoplasmic DNA, STING on the ER can rapidly dimerize and transfer from the ER to the nuclear peripheral bodies. Interestingly, TBK1 also aggregates into the nuclear peripheral bodies and forms the STING-TBK1 complex, which is essential for the activation of TBK1 (Fig. 3c).^261,262^

Currently, various polyubiquitinations of STING have been identified, including polyubiquitination of K63, K48, K11 and K27, all of which play important roles in the innate immune response against RNA and DNA infections. The different connections between these polyubiquitin chains not only broaden the functional spectrum of STING but also determine its strength and duration in regulating the expression of type I interferon genes. Under the stimulation of exogenous DNA, Trim56 induces K63 linkage ubiquitination of STING and promotes STING dimerization and recruitment to TBK1.^263^ In addition to Trim56, the E3 ligase Trim32 promotes the interaction between STING and TBK1 by catalyzing the K63-linked polyubiquitination of STING and finally increases the expression of STING-mediated interferon-β.^264^ To control cancer cells, HER2 also connects with STING and recruits AKT1 to directly phosphorylate TBK1, which prevents TBK1 K63-linked ubiquitination.^265^ Additionally, K48 polyubiquitination also inhibits signal transduction by promoting the degradation of STING. In detail, under the stimulation of DNA or RNA, RNF5 catalyzes the K48 polyubiquitination of STING at K150 and K48. This modification serves as a proteolytic signal by targeting STING for degradation via the 26S proteasome.^266^ The E3 ligase RNF26 localized on the ER catalyzes the ubiquitination of the K11 linkage of STING. In the early stage of viral infection, the K11 polyubiquitin chain catalyzed by RNF26 competes with the K48 ubiquitination of STING, prevents the RNF5-mediated degradation of STING, and increases the expression of type I interferon, whereas in the late stage of a viral infection, RNF26 inhibits the expression of type I interferon by promoting lysosomal degradation of IRF3.^267^ In addition, K27-linked polyubiquitination of STING induced by autocrine motility factor receptor (AMFR) works as a molecular platform to recruit TBK1 and promotes the translocation of TBK1 to the nuclear peripheral bodies (Fig. 3c).^268^

Modification of the K27- and K63-linked ubiquitination chains of STING activates anti-DNA viral effects in cells. USP21 can interact directly with STING and remove the K27 and K63 Ub chains on STING, thereby inhibiting the production of type I interferons. In the late stage of herpes simplex virus 1 (HSV-1) infection, protein kinase p38 phosphorylates USP21 and recruits it to bind to STING. Inhibiting the activity of p38 in mice blocks the binding of USP21 to STING, which in turn protects mice from an HSV-1 infection by inhibiting the production of type I interferons. Additionally, in USP21 knockout mice, resistance to DNA viruses was enhanced (Fig. 3c).^269^

## Ubiquitination in CSC stemness maintenance

The “stemness” state of stem cells is the key ability to self-renew and differentiate into the germline. Stem cells can be found in adult and embryonic tissues and play an extremely important role in cell regeneration, growth and embryonic development. CSCs are a subpopulation of tumor masses with pluripotent tumorigenesis, metastasis dissemination, drug resistance and cancer recurrence.^270^ In CSCs, a fine-tuning circuit consisting of a core set of transcription factors regulates stemness-specific gene expression profiles, including the core stem cell regulator triplet, Oct4, Sox2 and Nanog.^271–273^ In addition, some signaling pathways, including the Hippo and Wnt signaling pathways, also participate in CSC stemness maintenance. Ubiquitination plays an important role in CSC characteristics, such as self-renewal, maintenance, differentiation and tumorigenesis. By comparing the protein expression and ubiquitination levels between pluripotent and differentiated stem cells via quantitative proteomics, Iannis surprisingly found the ubiquitination of core transcription factors, which included Nanog, Oct4 and Sox2, indicating the crucial roles of the ubiquitination-mediated transcriptional regulatory network in maintaining the stemness and pluripotency of stem cells.^274^ This section focuses on the recent progress in the ubiquitination-mediated transcriptional regulatory network and signaling pathways in maintaining the stemness and pluripotency of stem cells.

## Nanog ubiquitination

**Fig. 4:**
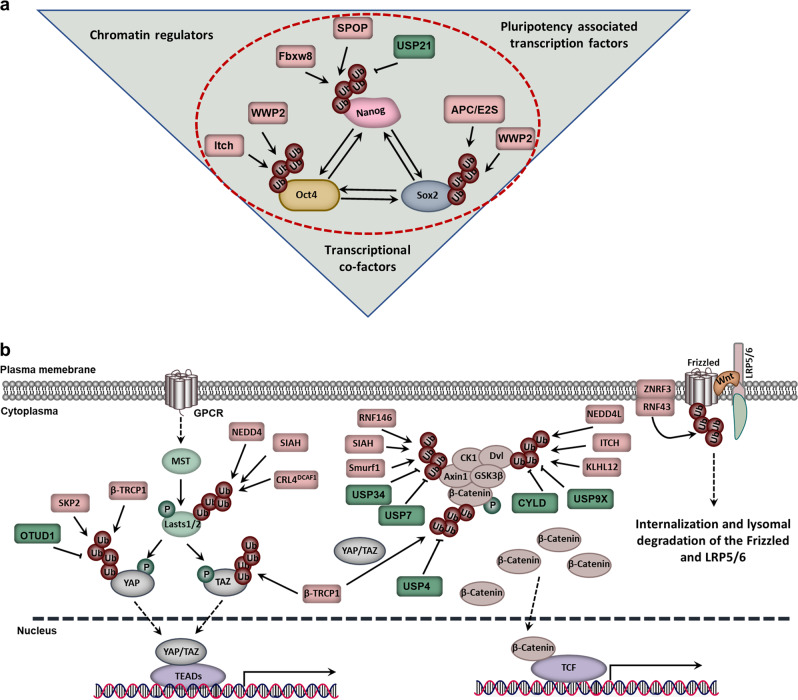
Ubiquitination in cancer stem cell (CSC) stemness maintenance. **a** The ubiquitination-mediated regulation of the transcriptional regulatory network in maintaining the stemness of stem cells. **b** Ubiquitination in the Wnt and HIPPO signaling pathways

As the key transcription factor for maintaining stem cell pluripotency and promoting somatic cell reprogramming, Nanog is mainly regulated by its allele, transcription factors and PTM in stem cells. Nanog contains a degradation determinant PEST sequence with a very short half-life. However, the regulatory mechanism of Nanog stability was unclear until 2014. Researchers identified that ERK1 phosphorylated the Ser52 of Nanog and promoted its interaction with Fbxw8, which played an important role in Nanog's proteasome pathway degradation and the differentiation of stem cells (Fig. 4a).^275^

In addition to Fbxw8, another investigation proposed that SPOP, the Cullin3-dependent E3 ligase, could also mediate Nanog degradation. SPOP contains a BTB domain linked to Cullin3 and a MATH domain that specifically recognizes and binds to substrates. A variety of biological processes, including cell proliferation, apoptosis and cell senescence, are regulated by SPOP by degrading a variety of substrates, such as AR, DEK, ERG, SRC3, DAXX and SENP7.^276–281^ Two independent studies revealed that the Pin1 or AMPK-BRAF signaling pathway phosphorylated Nanog-Ser68; then, the modified Nanog was recognized and polyubiquitinated by SPOP and finally degraded via the proteasome (Fig. 4a).^282,283^

As ubiquitination is a reversible process, Nanog may also be regulated by deubiquitinating enzymes during stem cell or somatic cell reprogramming. To screen the DUB of Nanog, the deubiquitinating enzyme USP21 was identified by the efficient dual-luciferase reporter assay system. USP21 could significantly enhance the stability of Nanog and then maintain the self-renewal of stem cells. The interaction between USP21 and Nanog could be blocked by ERK-mediated phosphorylation at position 539 of USP21, which subsequently promoted the differentiation of stem cells by downregulating the stability of Nanog.^284,285^ Additionally, USP21 was also reported to maintain the self-renewal of embryonic stem cells by stabilizing Nanog (Fig. 4a).^285,286^

### Oct4 ubiquitination

As a member of the POU transcription factor family, Oct4 plays a key role in maintaining the stemness and pluripotency of stem cells.^287^ In stem cells, the protein level of Oct4 is accurately regulated, and the abnormal expression of Oct4 is the main cause of somatic cell cloning failure. In terms of the regulation of Oct4, the UPS plays an important role. WWP2, an E3 ligase of the HECT family, interacts directly with Oct4 and mediates its proteasome degradation by promoting its ubiquitination.^288,289^ Additionally, the E3 ligase ITCH can also interact with Oct4, promote its ubiquitination and then regulate Oct4 transcription activation; however, ITCH cannot mediate the degradation of Oct4 (Fig. 4a).^288^ The different functions of the two E3 ligases for Oct4 in stem cells suggest that the same substrate can be regulated by different E3 ligases. In addition, ERK1 can phosphorylate Ser111 of Oct4, induce its ubiquitination and promote its degradation and cytoplasm location.^289^

### Sox2 ubiquitination

Similar to Oct4 and Nanog, the protein level of the core transcription factor Sox2 is also regulated by the UPS in stem cells.^290^ In 2014, the methylation enzyme Set7 was found to induce the monomethylation of Sox2. It not only inhibited the expression of Sox2 but also promoted the ubiquitination of Sox2 by facilitating the interaction between WWP2 and Sox2. Thus, Set7 can mediate the degradation of Sox2 and stem cell differentiation. In contrast, AKT phosphorylates Sox2 and inhibits the Set7-mediated methylation of Sox2, thereby inhibiting the ubiquitination of Sox2 and maintaining the self-renewal of stem cells, suggesting that Sox2 is precisely regulated by PTM to maintain stem cell pluripotency and direct differentiation.^291^ As an important regulator of stem cell self-renewal, Sox2 can also interact with APC and Ube2s directly, which mediates the degradation of Sox2 by promoting K11 linkage ubiquitination at the Lys123 of Sox2 (Fig. 4a).^292–295^

In addition, USP7 is able to maintain stem cell self-renewal by inhibiting the E3 ubiquitin ligase β-TrCP1-mediated ubiquitination of REST, a stemness transcription factor that plays an important role in neural differentiation (Fig. 4a).^296^ In addition to USP7, many deubiquitinating enzymes have been identified in transcriptional regulation in stem cells based on genome-wide localization analysis. For example, USP10, USP16, USP3, USP37 and USP44 are reported to bind to the promoter of Nanog; USP44 is capable of binding to the promoter of Oct4; and USP22, USP25, USP44 and USP49 are proven to bind to the promoter of Sox2.^273^

### Ubiquitination in the Wnt signaling pathway

The Wnt signaling pathway is an evolutionarily conserved signaling pathway that is critical in regulating CSCs.^297^ In the absence of Wnt on the cell surface, the “destructive complex” of β-catenin, a multisubunit complex consisting of four proteins, GSK3β, Axin, casein kinase 1α (CK1α) and adenomatous polyposis coli (APC), can bind to β-catenin and promote its phosphorylation at the N-terminus via CK1α and GSK3β. β-TrCP1 can recognize phosphorylated β-catenin and promote its ubiquitination and degradation, thereby negatively regulating the Wnt signaling pathway.^298–300^ As a ligand, Wnt bridges the Frizzled-LRP5/6 protein and phosphorylates LRP5/6 through CK1a. Subsequently, it recruits the “destructive complex” to the cell membrane, inhibits the phosphorylation and ubiquitination of β-catenin and promotes its accumulation in the nucleus to regulate CSC stemness maintenance.^301,302^

In addition to the degradation of β-catenin, the internalization and lysosomal degradation of Frizzled-LRP5/6 can be regulated by the E3 ligases ZNRF3- and RNF43-mediated ubiquitination. It is also an important way to negatively regulate Wnt signaling.^303–306^ As a feedback regulator of Wnt signaling, inactivation of RNF43 and ZNRF3 leads to a significant expansion of the crypt proliferation region and promotes tumorigenesis. Moreover, RNF43 inactivating mutations can be found in various cancers.^307,308^

Axin is a key scaffold protein in the “destructive complex”, and its regulation is associated with the Wnt signaling pathway. For example, the E3 ligase RNF146 promotes ubiquitination-mediated proteasomal degradation of Axin on the basis of the PARsylation of Axin,^309,310^ while SIAH binds to Axin and mediates its degradation, which amplifies the feedback regulation of the Wnt signaling pathway.^311^ Unlike the degradation signal, SMURF1 inhibits its interaction with LRP5/6 by mediating the K29-linked polyubiquitination of Axin and negatively regulates the Wnt signaling pathway.^312^

Notably, UPS also regulates the ubiquitination of different components in the Wnt signaling pathway. For example, Nedd4L, ITCH and KLHL12 are able to negatively regulate the Wnt signaling pathway by targeting Disheveled (Dvl) degradation.^313–315^ In addition, the deubiquitinating enzymes USP34/USP7, CYLD/USP9X and USP4 can bind to Axin, Dvl and β-catenin, respectively, thereby promoting the nuclear localization of β-catenin and the Wnt signaling pathway by inhibiting their ubiquitination.^316–320^

### Ubiquitination in the Hippo-YAP signaling pathway

In mammals, the Hippo signaling pathway can also maintain CSC stemness, regulate cell growth, control the size of organs and take part in tumorigenesis.^321,322^ Ubiquitination also plays an important role in regulating the Hippo signaling pathway, with a variety of E3 ligases being identified. For example, CRL4DCAF1 negatively regulates the Hippo signaling pathway by ubiquitinating and degrading Lats1 while promoting the monoubiquitination of Lats2 and inhibiting its activity.^323^ Similarly, the stability of Last2 is also regulated by the E3 ligase SIAH2. SIAH2, an important regulator of the HIF signaling pathway, degrades PHD3/1 by ubiquitination in a hypoxic environment. In turn, the activation of the Hippo signaling pathway also controls the stability of HIF1α and the HIF1α signaling pathway (Fig. 4b).^194,324^ This part of the work highlights the important roles of the hypoxic environment in regulating the ubiquitination of the Hippo signaling pathway.

YAP/TAZ is the key component in the Hippo signaling pathway. The stability of YAP/TAZ is also controlled by PTM. For example, phosphorylated YAP is recognized and ubiquitinated by the E3 ligase β-TrCP1.^325^ The CK1ε-mediated phosphorylation of TAZ is recognized by β-TrCP1 and promotes the Κ48-linkage ubiquitination of TAZ. The ubiquitination of TAZ mediates its entrance into the proteasome for degradation (Fig. 4b).^326^ Unlike K48 linkage ubiquitination, a recent report indicated that the E3 ligase SKP2 induces the nonproteolytic K63 linkage ubiquitination of YAP and leads to its nuclear localization and interaction with the nuclear binding partner TEAD. In this process, OTUD1 could remove the K63 linkage ubiquitination of YAP and negatively regulate transcriptional activity and cell growth (Fig. 4b).^327^

The Wnt signaling pathway, associated with CSC stemness maintenance, also plays an important role in regulating the stability and degradation of TAZ. Phosphorylated β-catenin can serve as a platform for TAZ and β-TrCP1 and promotes TAZ degradation by β-TrCP1.^328^ Moreover, YAP/TAZ is essential for β-TrCP1 recruitment to the APC complex and β-catenin inactivation. Under Wnt OFF conditions, YAP/TAZ is sequestered in the APC complex by binding to Axin1 and then recruiting β-TrCP1 to degrade β-catenin. Under Wnt ON conditions, YAP/TAZ is dissociated from Axin1 and accumulates in the nucleus to regulate the Wnt/β-catenin signaling pathway.^329^ In addition to the Ub ligases reported above, the K48 linkage polyubiquitination mediated by Nedd4 also functions as a proteolytic signal and degrades WW45 and Last1/2 via the proteasome (Fig. 4b).^330^ Taken together, these clues indicate that ubiquitination can regulate the Hippo and Wnt signaling pathways by controlling the stability of different substrates.

## Cancer therapeutic strategy via targeting the UPS

As mentioned above, the UPS plays an essential role in protein degradation and fundamental cellular process regulation.^331,332^ Genetic alterations, abnormal expression or dysfunction of the UPS often lead to human pathogenesis, especially cancer. Thus, these components can serve as potential drug targets for therapeutic strategies against cancer.^8^ Currently, many small molecule inhibitors have been developed that target different components of the UPS, which include the proteasome, E3 ligases, E1 enzymes, E2 enzymes and DUBs, and their therapeutic effects are gradually being tested.^333^

### Targeting the proteasome activity

Among all UPS components, only the proteasome has been successfully exploited as a therapeutic target for the clinical treatment of cancer. Tangible success has been achieved using proteasome inhibitors (PIs), such as bortezomib, carfilzomib, oprozomib and ixazomib (Fig. 4b).^334,335^ Under normal physiological conditions, selective tagging of proteins with Ub is targeted to the proteasome and results in proteasome-mediated proteolysis.^336^ The proteasome exhibits three distinct activities, namely, chymotrypsin-like, trypsin-like and caspase-like activities. Its alterations are found in various human diseases. In tumorigenesis, proteasome abnormalities are not observed; thus, the function of the proteasome in tumor cells may be on the basis of their own needs.^11^

The boronic acid derivative bortezomib (Velcade, Millennium Pharmaceuticals), a unique first-in-class compound, can slowly and reversibly block chymotrypsin-like and decrease trypsin-like and caspase-like activities of the 20S proteasome.^337^ Previous studies have proven that bortezomib can inhibit proliferation and induce cell apoptosis by blocking the NF-κB pathway, activating the c-Jun/AP-1 pathway and increasing cyclin-CDK inhibitors (p21 and p27) in various tumor cell lines, such as squamous cell carcinoma, multiple myeloma (MM), mantle cell lymphoma (MCL), hepatocellular carcinoma and non-small-cell lung cancer (NSCLC).^338–343^ In the clinic, it is the first approved PI by the U.S. Food and Drug Administration (FDA) for relapsed MM^344^ and MCL.^345^ Later, it was expanded for use in patients with NSCLC and pancreatic cancer.^346^ Despite its promising results, some off-target and adverse effects, such as fatigue, asthenia, thrombocytopenia, peripheral neuropathy and gastrointestinal symptoms, also limit its application.^347,348^ The off-target effects may lead to dose-limiting toxicity and subsequently result in permanent nerve damage to the extremities, called bortezomib-induced peripheral neuropathy (BIPN).^349^ Moreover, bortezomib resistance may occur within an average of 1 year, especially for solid tumors.^350–352^ The resistance mechanism includes an enhanced aggresome-autophagy pathway, increased expression of proinflammatory macrophages, alterations in apoptotic signaling and decreased ER stress response.^353,354^

**Fig. 5:**
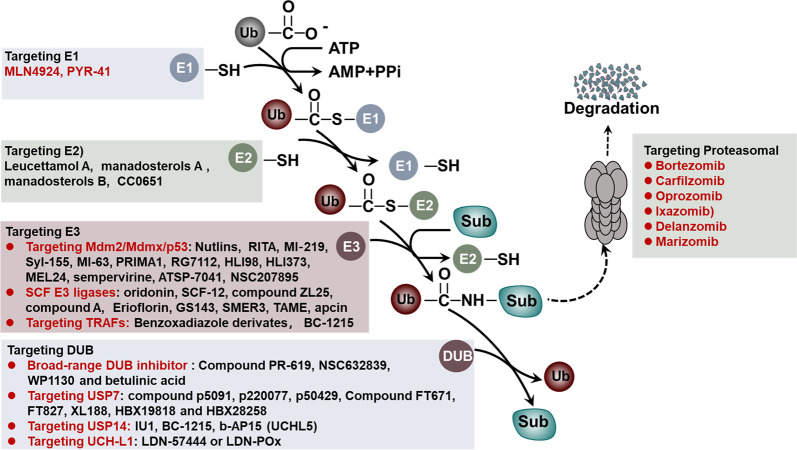
Cancer therapeutic strategy by targeting the UPS

Another approved PI for relapsed or refractory MM is carfilzomib (PR-171; Kyprolis; Onyx Pharmaceutical), a second-in-class PI (Fig. 5).^355^ Similar to bortezomib, carfilzomib also inhibits the chymotrypsin-like activity of the 20S proteasome. However, unlike bortezomib, the activity of carfilzomib is irreversible. In addition, carfilzomib is more effective than bortezomib in acute myeloid leukemia (AML) cells by inducing apoptosis and inhibiting proliferative activity.^356^ It also shows improved safety in terms of peripheral neurotoxicity and maintains its cytotoxic potential in bortezomib-resistant cell lines.^357^ Due to the good tolerance and promising efficacy for MM in phase I and II clinical trials, carfilzomib was approved by the FDA for the treatment of relapsed MM patients who experience disease progression within 60 days after the treatment of bortezomib and immunomodulatory drugs.^358^ Carfilzomib treatment can also cause adverse effects, such as cardiovascular complications (hypertension, heart failure), hematologic complications (thrombocytopenia, anemia), gastrointestinal complications (diarrhea, nausea/vomiting) and systemic symptoms (fever, fatigue). Therefore, its treatment should also be monitored carefully^359,360^

As a new generation of PIs, oprozomib (ONX0912; PR-047) is designed as a tripeptide analog of carfilzomib (Table 1).^361^ In contrast to intravenously administered bortezomib and carfilzomib, oprozomib has better oral bioavailability and is suitable for oral administration. It also has a similar antitumor activity, potency and selectivity as carfilzomib in MM and can be used to treat bortezomib-, dexamethasone- or lenalidomide-resistant MM.^362^ In the treatment of solid tumors, it induces cell apoptosis by upregulating proapoptotic Bik and Mcl-1.^363^ However, due to oral administration, oprozomib has a high rate of gastrointestinal toxicities and unstable pharmacokinetics.^364^

**Table 1:** Selected compounds targeting the UPS

Target	Compounds	Molecular mechanisms	Status
20S proteasome	Bortezomib^507^	Inhibition of proteasome-mediated proteolysis, which may lead to cell cycle arrest or apoptosis or inhibit the tumor growth.	FDA approved for MM, MCL, NSCLC and PaCa
	Carfilzomib^508^	Inhibition of proteasome-mediated proteolysis, which may lead to cell cycle arrest or apoptosis or inhibit the tumor growth.	FDA approved for relapsed and refractory MM
	MLN9708^509^	A second-generation small-molecule proteasome inhibitor that displays antitumor activity in a variety of mouse models of HM.	Phase III for MM
	Marizomib^371^	A novel proteasome inhibitor that exhibits effects in patients with refractory and relapsed MM.	Phase III for Glioblastoma
CRBN	Thalidomide^510,511^	Binds to CRBN and suppresses its activity.	FDA approved for MM
	Lenalidomide, Pomalidomide^512–514^	Binds to CRBN and suppresses its activity.	FDA approved for refractory MM
Mdm2	PRIMA^387,515,516^	Restores transcriptional activity of unfolded wild-type or mutant p53.	FDA approved for LiCa and PaCa
	Serdemetan^517,518^	Increases p53 levels and inhibits proliferation, formation of the capillary tube and migration of HMEC-1 cells.	Phase I for solid tumor
	RG7112^519^	Increases p53 levels and transcriptional activation of p53 target genes.	Phase I for HM
	RG7388^61,520,521^	Increases p53 levels and signaling and suppresses neuroblastoma cell growth.	Preclinical/research
	RITA^383^	A small molecule inhibitor preventing the interaction between p53 and Mdm2 in the A-498 and TK-10 cell lines.	Preclinical/research
	HLI373^389,397^	Increases p53 levels through inhibiting Hdm2-mediated ubiquitination in U2OS cells.	Preclinical/research
	MEL23^390^	Increases p53 levels in U2OS, RKO and HCT116 cultures, and shown to induce RKO and MEF cell death.	Preclinical/research
	Nutlin-3a^522^	Inhibits the growth of HM, GBM and AML cells by activating the p53-dependent apoptotic pathway.	Preclinical/research
	HLI98^388^	Activates p53 signaling and inhibits the tumor cell growth.	Preclinical/research
MdmX	SJ-172550^523^	Inhibits the MDMX-p53 interaction in cultured retinoblastoma cells.	Preclinical/research
	NSC207895^395^	Inhibits MDMX expression in MCF-7 cells.	Preclinical/research
	FL118^524^	Induces p53-dependent senescence in colorectal cancer cells.	Preclinical/research
FBW7	SCF-I2^399^	Inhibits the ubiquitination of substrates via inhibiting SCF^Cdc4^.	Preclinical/research
SKP2	Compound #25^400^	Inhibits the activation of Skp2 and exhibits antitumor activity in PC3-induced tumor xenografts.	Preclinical/research
APC	Apcin^409^	Blockades the substrate proteolysis and impedes cell mitotic exit via inhibiting Cdc20.	Preclinical/research
	TAME^408^	Prevents the activation of APC by Cdc20 and Cdh1.	Preclinical/research
β-TrCP1	Erioflorin^405^	Inhibits the NF-κB signaling pathway by decreasing the IκB ubiquitination.	Preclinical/research
	GS143^406^	Inhibits the NF-κB signaling pathway by decreasing the IκB ubiquitination.	Preclinical/research
UBA1	MLN7243^417^	Causes the depletion of cellular ubiquitin conjugates and shows antitumor activity in primary human xenograft.	Phase I for advanced malignant solid tumors
NAE	MLN4924^419^	Blocks the interaction between NAE and NEDD8 by forming an irreversible covalent adduct with NEDD8.	Phase III for higher-risk MDS, CMML, AML
USP1	Pimozide^435^	Decreases GBM in xenograft models.	Phase I/II for GBM
USP7	p5091^434,440,441^	Induces apoptosis in MM cells, which are resistant to conventional and bortezomib therapies in mouse tumor model studies.	Preclinical/research
	p22077, p50429^441^	Covalently modifies the catalytic cysteine of USP7.	Preclinical/research
	FT671, FT827^442^	Destabilizes USP7 substrates and results in the inhibition of tumor growth in mice.	Preclinical/research
	HBX19,818, HBX28,258^444^	Binds in USP7 active site and shows effects on cell proliferation, apoptosis and cell cycle.	Preclinical/research
USP14	XL188^443^	A noncovalent active-site inhibitor that promotes the accumulation of p53 and p21.	Preclinical/research
	GNE-6640, GNE-6776^525^	A noncovalent active-site inhibitor of USP14 that induces tumor cell death.	Preclinical/research
	IU1^450,451^	Enhances the degradation of several proteasome substrates that have been implicated in neurodegenerative disease.	Preclinical/research
USP14, UCHL5	b-AP15^455^	Decreases viability in MM cell lines and patient MM cells, inhibits proliferation of MM cells via the downregulation of CDC25C, CDC2 and cyclin B1.	Preclinical/research

*MM* multiple myeloma, *MCL* mantle cell lymphoma, *NSCLC* non-small-cell lung cancer, *PaCa* pancreatic cancer, *HM* hematologic malignancies, *LiCa* liver cancer, *MDS* myelodysplastic syndrome, *CMML* chronic myelomonocytic leukemia, *AML* acute myelogenous leukemia, *GBM* glioblastoma

Ixazomib citrate (MLN9708) and its biologically active form ixazomib (MLN2238) are the first orally administered PIs with improved pharmacokinetic and pharmacodynamic profiles (Table 1).^365^ Like bortezomib, Ixazomib citrate is a boron-containing peptidic agent that reversibly targets the β5 proteasome subunit and inhibits chymotrypsin-like proteasome activity. In the clinic, ixazomib citrate is also recommended for the treatment of relapsed and refractory MM with a positive safety profile.^357,366,367^ Moreover, in a phase 3 trial (NCT02181413), ixazomib maintenance can prolong progression-free survival (PFS) and is regarded as an additional option for posttransplant maintenance therapy in patients with newly diagnosed MM.^368^ Delanzomib (CEP-18770) is also a reversible and orally bioavailable structural analog of bortezomib (Fig. 5). Although it overcomes the resistance of bortezomib to peripheral neuropathy, severe skin toxicity limits its clinical application.^369^

Marizomib (NPI-0052), a novel PI, can affect chymotrypsin-like, trypsin-like and caspase-like activities of the 20S proteasome (Table 1). It can overcome bortezomib resistance and exhibit broader anticancer activities, with a better therapeutic ratio.^370^ Additionally, marizomib exhibits synergistic effects with bortezomib and lenalidomide or pomalidomide and low-dose dexamethasone in patients with refractory and relapsed MM.^362,371^ In addition, marizomib has the ability to penetrate the blood–brain barrier and induce apoptosis in glioma cells with low toxicity on normal cells.^372^ Thus, it has been applied in newly diagnosed glioblastoma (NCT03345095). The side effects of marizomib are limited to gastrointestinal symptoms without neuropathy or other significant systemic toxicities.^373^

As a hierarchical biological system, the UPS displays multilevel regulation of protein ubiquitination (Table 2). The PI targets only the last step of the ubiquitination process and thus leads to the accumulation of upstream ubiquitinated proteins, which may lead to other side effects. For instance, BIPN occurrence is associated with the aggregation of Ub-laden proteins in the dorsal root ganglia, which may result in bortezomib dose reduction or even discontinuation.^352^ Thus, other agents targeting other aspects of the UPS should also be developed.

**Table 2:** Abbreviations

Full name	Abbreviation	Full name	Abbreviation
Ubiquitin proteasome system	UPS	Ubiquitin	Ub
Ub-activating enzyme	E1	Ub-conjugating enzyme	E2
Ub-ligating enzyme	E3	Cancer stem cells	CSCs
Deubiquitinases	DUBs	Posttranslational modification	PTM
Ring finger protein 152	RNF152	Ring between ring fingers	RBR
Skp1-cullin1-F-box	SCF	Murine double minute 2	Mdm2
Anaphase-promoting complex/cyclosome	APC/C	F-box/WD repeat-containing protein 7	FBXW7
Homologous to the E6-AP carboxyl terminus	HECT	S-phase kinase-associated protein 2	SKP2
CDC20-like protein 1	CDH1	Really interesting new gene	RING
E6-related protein	E6AP	HDM2 being the human enzyme	Hdm2
OTU domain-containing ubiquitin aldehyde-binding protein 1	OTUB1	Beta-transducin repeat containing protein 1	β-TrCP1
Ubiquitin-like	UBL	Nuclear factor-kappa-B	NF-κB
Parkinson’s disease	PD	ADP-ribosylated Ub	ADPr-Ub
Siderophore E	SidE	Phospho-ribosylated Ub	Pr-Ub
Guanosine exchange factor	GEF	NEDD8 activating enzyme	NAE
Vacuolar H+-ATPase	v-ATPase	Poly(ADP-ribose) polymerase 1	PARP1
Cylindromatosis	CYLD	TNF receptor-associated factor	TRAF
Von Hippel-Lindau	VHL	Tumor microenvironment	TME
Tetratricopeptide repeat domain 3	TTC3	WW domain-containing ubiquitin E3 ligase 2	WWP2
Adenylate-activated protein kinase	AMPK	Guanine nucleotide dissociation inhibitors	GDIs
Endoplasmic reticulum	ER	Tumor-draining lymph nodes	TDLNs
Toll-like receptor	TLR	Tumor-associated macrophages	TAM
RIG-like receptor	RLR	Lipopolysaccharide	LPS
Cancer-associated fibroblasts	CAFs	Melanoma differentiation-associated protein 5	MDA5
Retinoic acid-inducible gene 1	RIG-I	Laboratory of genetics and physiology 2	LGP2
Hypoxia-inducible factor-1α	HIF-1α	Linear ubiquitin chain assembly complex	LUBAC
Autocrine motility factor receptor	AMFR	PYD domains-containing protein 4	NLRP4
Antigen-presenting cells	APCs	Non-small-cell lung cancer	NSCLC
Herpes simplex virus type 1	HSV-1	Food and Drug Administration	FDA
Glycogen synthase kinase 3	GSK3β	COP9 signalosome	CSN
Axin/casein kinase 1α	CK1α	Small ubiquitin-related modifier	SUMO
Adenomatous polyposis coli	APC	Promyelocytic leukemia	PML
Disheveled	Dvl	Heterochromatin protein 1	HP1
Double-strand break	DSB	Otubain proteases	OTUs
Major histocompatibility complex I	MHC I	Homologous recombination	HR
Sentrin-specific protease 7	SENP7	PTEN-induced putative kinase 1	PINK1
Liver kinase B1	LKB1	GTPase-activating proteins	GAPs
Cullin-RING ubiquitin ligases	CRLs	Ataxia telangiectasia mutated	ATM
Protein inhibitor of activated STAT	PIAS	C-terminus of Hsc70 interacting protein	CHIP
Ras homolog enriched in brain	Rheb	Mechanistic target of rapamycin complex 1	mTORC1
Epidermal growth factor	EGF	Mammalian lethal with SEC13 protein 8	mLST8
DEP domain-containing mTOR-interacting protein	DEPTOR	Calcium/calmodulin-dependent protein kinase kinase 2	CaMKK2
Progressive multifocal leukoencephalopathy	PML	OTU deubiquitinase 7B	OTUD7B
TGF-β-activated kinase 1	TAK1	Receptor-interacting serine/threonine-protein kinase 1	RIPK1
IκB kinase	IKK	TANK-binding kinase 1	TBK1
TRAF family member-associated NFKB activator	TANK	TIR-domain-containing adapter-inducing interferon-β	TRIF
Mitochondrial antiviral signaling	MAVS	NF-κB essential modulator	NEMO
Proliferating cell nuclear antigen	PCNA	Proteasome inhibitors	PIs
Multiple myeloma	MM	Acute myeloid leukemia	AML
Mantle cell lymphoma	MCL	Progression-free survival	PFS
Bortezomib-induced peripheral neuropathy	BIPN	Reactivation of p53 and induction of tumor cell apoptosis	RITA
Inhibitor of nuclear factor-κB	IκB	Mesencephalic astrocyte-derived neurotrophic factor	MANF
F-box proteins	FBPs	Ubiquitin-specific proteases	USPs
Machado–Joseph disease protein domain proteases	MJD	Ubiquitin carboxyl-terminal hydrolases	UCHs
Small-molecule enhancers of rapamycin 3	SMER3	JAMM/MPN domain-associated metallopeptidases	JAMMs
*N*-4-Tosyl-l-arginine methyl ester	TAME	Monocyte chemotactic protein-induced protein	MCPIP

### Targeting E3 enzymes

As the most important components of the Ub conjugation machinery, E3 ligases mediate degradation with high substrate specificity.^374^ Thus, targeting the active site of E3 enzymes or their interactions with substrates offers promising options for developing drugs with fewer side effects.

#### Mdm2/Mdmx/p53

As noted above, the high-profile target is Mdm2/Hdm2, which binds to the amino terminus of p53 and mediates the degradation of p53 by Ub-dependent mechanisms.^375^ Due to the critical role of p53, many efforts have been made to find an antagonist of the E3 ligase Mdm2/Hdm2 (Fig. 5).

The crystal structure reveals that the N-terminal domain of Mdm2 complexes with a small peptide of p53, including three critical amino acids: Phe19, Trp23 and Leu26.^376^ Moreover, the peptide has a far higher affinity for Mdm2 and can serve as the “druggable” pocket.^377^ The first nonpeptidic molecule demonstrated to interrupt the p53–Mdm2 interface is 4,5-dihydroimidazoline (nutlin; Roche).^378^ Nutlin and its derivatives (for example, nutlin-3a, also known as RG7112, and RG7388) are the most successful chemical compounds identified by high-throughput screening (Table 1). Structurally, they are mimics of p53 and specifically bind to the pocket on Mdm2, thereby disrupting the protein–protein interface.^379^ Nutlins have been shown to inhibit the growth of hematological malignancies, glioblastomas and AML cells by activating the p53-dependent apoptotic pathway.^378,380,381^ In addition, they do not induce apoptosis in normal cells.^382^ Although the primary results of nutlins are promising, their shortcomings have recently been uncovered. Nutlins can only be used in tumor cells with wild-type p53, whereas they are insensitive to p53-deleted or p53-mutated cells. Moreover, it has been reported that nutlins are not specific for p53 and may compete with other proteins for Mdm2 binding. The same shortcomings are also found in RITA (reactivation of p53 and induction of tumor cell apoptosis), a small molecule inhibitor preventing the interaction between p53 and Mdm2 both in vitro and in vivo.^383^ Guided by the interactions among the p53 peptide, nutlin and Mdm2, a new class of inhibitors was developed and includes MI-219,^384^ SyI-155,^385^ MI-63,^386^ PRIMA1^387^ and RG7112 (Table 1).^375^ For example, MI-219 is an orally available compound with a subnanomolar affinity for Mdm2 and can increase the expression of p53 and p53-targeted genes.^384^

In addition, other small molecules, which include HLI98,^388^ HLI373,^389^ MEL24 (ref. ^390^) and the natural product sempervirine (Table 1),^391^ can also target the E3 ligase activity of Mdm2 directly and prevent the degradation of p53. HLI98 has been shown to activate p53 signaling and inhibit tumor cell growth in a p53-dependent manner. HLI373 is the highly water-soluble derivative of HLI98, and it has a greater potential to be a cancer therapeutic agent due to this characteristic.^389^

Although Mdmx (murine/human double minute X) has no ubiquitination activity, it can either bind to the N-terminus of p53 and inactivate it directly or ubiquitinate p53 by heterodimerizing with Mdm2.^375^ Nutlin-3 exhibits poor inhibition of Mdmx–p53 interactions, and there is a need for dual inhibitors of both Mdm2 and Mdmx due to the high expression of Mdmx in some cancers.^392,393^ ATSP-7041, a dual inhibitor, can induce p53-dependent tumor growth suppression.^394^ NSC207895, targeting Mdmx specifically, can act additively with nutlin-3a to activate p53 and induce apoptosis (Fig. 5).^395^

#### SCF E3 ligases

As mentioned before, SCF E3 ligases are also a well-known family of E3 ligases and includes FBXW7, SKP2 and β-TrCP1.^396^ Since FBPs are responsible for the specificity of SCFs, many small molecules have been designed to target them and may provide attractive therapeutic agents.^63^

Mutations in FBW7 and its targets often block the degradation of these oncogenic substrates (c-Myc, c-Jun and Notch) and subsequently promote tumorigenesis.^397^ Some efforts have been made to develop agonists for the FBW7 E3 ligase complex. The natural compound oridonin promotes FBW7-mediated proteasomal degradation of c-Myc, thereby inducing apoptosis in leukemia and lymphoma cells.^398^ By small molecule screening, SCF-12 allosterically inhibits the recognition of the substrate FBP Cdc4 (a homolog of FBW7 in yeast) but not its human ortholog FBXW7 (Table 1).^399^

For SKP2, compound ZL25 can inhibit SKP2 directly and subsequently result in cancer cell senescence in a p53-independent way.^400^ Compound A, another SKP2 inhibitor, was also found to induce cell cycle arrest and cell death in a p27-dependent manner. The inhibition blocks SCF–SKP2 complex formation (Fig. 5).^401^

β-TrCP1 can ubiquitinate the phosphorylated inhibitor of nuclear factor-κB (IκB) and lead to its degradation by the proteasomal pathway.^402,403^ A small phosphopeptide agonist has been designed to target the IκB Ub ligase, and this peptide can inhibit IκB degradation in TNF-stimulated HeLa cells.^404^ Erioflorin, an inhibitor of β-TrCP1, has been shown to suppress the activity of NF-κB and decelerate cell proliferation in various cancer cells by stabilizing the tumor suppressor PDCD4 (Fig. 5).^405^ GS143, another inhibitor of β-TrCP1, can also inhibit the NF-κB signaling pathway by decreasing IκB ubiquitination (Fig. 5).^406^

Generally, the FBP MET30, a member of the SCF E3-ligase family, regulates various cellular processes, including cell proliferation, transcription and immune response.^407^ By a yeast-based screen, small-molecule enhancers of rapamycin 3 (SMER3) were found to directly bind to MET30 and block its Ub ligase (Fig. 5). In addition, APC/C initiates the metaphase–anaphase transition and mitotic exit by targeting proteins such as securin and cyclin B1 for Ub-dependent destruction by the proteasome. CDC20 binds to the APC/C complex and induces its activation in mitosis. N-4-Tosyl-l-arginine methyl ester (TAME) binds to the APC E3 ligase and inhibits its activation by targeting both CDH1 and CDC20 and inducing mitotic arrest.^408^ Another small molecule called apcin (APC inhibitor) binds directly to CDC20, competitively inhibits the ubiquitination of D-box-containing substrates, blocks mitotic exit, and then induces tumor cell death (Table 1).^409^

#### VHL E3 Ub Ligase

Mutations in the VHL E3 ligase often result in tumors, especially renal cell carcinoma.^410^ Its mutations can block the degradation of HIF and eventually lead to high vascularity and promote tumor growth even under normal oxygen conditions. A small molecule was generated by Buckley et al. to target the VHL E3 ligase protein and mimic the binding mode of HIF-1α, which might provide a novel therapeutic strategy for anemia and ischemia.^411^

### TRAFs

TRAF6, another member of the RING-domain family, is mentioned above to induce the K63-linked polyubiquitination and activation of IκappaB kinase (IκK), which in turn promotes the activation of downstream NF-κB signaling. Benzoxadiazole derivatives inhibiting TRAF6 can block the proliferation of lung and prostate cancer cells.^412^ BC-1215, an inhibitor of the FBP Fbxo3, degrades TRAF adapter proteins by inhibiting the degradation of Fbxl2 and then blocks inflammation and tumorigenesis (Fig. 5).^413–416^

### Targeting the E1 enzyme

The E1 enzyme is responsible for activating Ub molecules in the protein degradation process and plays an important role in tumorigenesis. Currently, many efforts have been made to explore compounds targeting the E1 enzyme. The adenosine sulfamate analogs, MLN7243 and MLN4924, have been reported as UBA1 and NAE inhibitors, respectively, and both are currently used in Phase I/II and Phase I clinical trials (Table 1).^417–419^ The latter can form an irreversible covalent adduct with NEDD8 and block the formation of thioester bonds between NAE and NEDD8. This process inhibits the neddylation of cullins and leads to the accumulation of cullin-mediated degradation of proteins such as p21, p27 and IκBα.^420^ Moreover, experimental inhibitors of E1 have also been developed.^421^ PYR-41 (4[4-3,5-dioxo-pyrazolidin-1-yl]-benzoic acid ethyl ester), an irreversible Ub E1, can inhibit E1 and block the initiation of ubiquitination (Fig. 5). It inhibits the degradation of p53 and promotes apoptotic cell death in a p53-dependent manner. In addition, it also controls inflammation by inactivating NF-κB and inhibiting the expression of cytokines, chemokines and inflammatory mediators.^422^

### Targeting E2 enzymes

E2 enzymes mainly mediate the conjugation of Ub to substrates. Currently, some efforts have been made to identify inhibitors that prevent the interaction between E1s and E2s or E2s and E3s. For instance, Leucettamol A, isolated from a marine sponge, *Leucetta aff. microrhaphis*, can inhibit the Ubc13–Uev1A interaction and block the formation of their complex (Fig. 5).^423^ With the same target of Ubc13–Uev1A, manadosterols A and B, isolated from the marine sponge *Lissodendoryx fibrosa manadosterols*, are also identified. They are regarded as the second and third natural compounds against the Ubc13–Uev1A interaction. Additionally, they are more potent than the abovementioned Leucettamol A (Fig. 5).^424^

CC0651, a small-molecule selective allosteric site inhibitor of the E2 enzyme hCdc34, can block the ubiquitination and degradation of p27 and then inhibit tumor cell proliferation (Fig. 5).^425^ In addition, small-molecule microarray-based screening has also been successfully used to identify the inhibitor of the SUMO E2 enzyme Ubc9, and a few small-molecule inhibitors have been found.^426^

### Targeting DUB activity

As mentioned, ubiquitination is a dynamic and reversible process, and DUBs mediate the removal and processing of Ub or polyubiquitin chains from ubiquitinated proteins.^427^ Many DUBs have been found to participate in various events during the cell cycle progression, genomic instability regulation and tumorigenesis processes.^428^ As such, a number of DUB inhibitors have been developed ranging from broad-spectrum inhibitors to specific inhibitors and identified as potential anticancer agents.^7,429–431^

As broad-spectrum inhibitors, compounds G5 and F6 were identified by a cell-level drug screening.^432^ They are chalcone DUB inhibitors and are reported to induce Bcl-2-independent apoptosis.^432,433^ By activity-based chemical proteomics, compound PR-619 was identified as a broad-range DUB inhibitor (Fig. 5).^434^ Another identified broad-spectrum DUB inhibitor is NSC632839. It can target USP2 and USP7 and trigger apoptotic cell death in cancer cell lines (Fig. 5).^432^ Pimozide, a specific USP1 inhibitor, can block glioma stem cell maintenance and radioresistance (Table 1).^435^ WP1130, a small-molecule compound, also inhibits the activity of several DUBs, including USP9x, USP5, USP14, UCHL5 and UCH37. Furthermore, it downregulates the anti-apoptotic protein MCL-1 and upregulates the proapoptotic protein p53, leading to anti-tumor activity.^436^ As a natural product, betulinic acid can be isolated from a variety of plants, including the Betula/birch tree. Recently, it was reported to be a nonselective DUB inhibitor and induce the loss of transmembrane potential and cancer cell apoptosis.^437,438^ However, as unspecific DUB inhibitors, these broad-spectrum inhibitors may amplify their biological effects and unspecific toxicity, including (1) accumulation of polyubiquitinated proteins or unanchored polyubiquitin chains; (2) accumulation of misfolded proteins; (3) reduction in the individual DUB activities; and (4) aberrant biological activities of DUB-regulated oncoproteins.^439^ Thus, specific DUB inhibitors are recommended for clinical application.

Due to the important roles of USP7 in controlling p53 stability, it is the famous USP target for drug development. Many small-molecule antagonists of USP7 have been developed. For example, compounds p5091, p220077 and p50429 have been reported to enhance the ubiquitination and degradation of Mdm2 and induce apoptosis of bortezomib-resistant MM cells^434,440,441^; in addition, compounds FT671 and FT827 were identified by a cocrystal structure and proved to target a dynamic pocket near the catalytic center of the autoinhibited apo form of USP7 (Table 1).^442^ Along with FT671, XL188 designed by the DUB costructure can destabilize USP7 substrates, increase the expression of p53 and p53 downstream target genes, including the tumor suppressor p21, and subsequently inhibit tumor growth.^442,443^ A structural class of small molecules represented by HBX 19,818 and HBX 28,258, as well as P22077 and P50429, have been identified by biochemical assays and activity-based protein profiling in living systems to specifically inhibit USP7.^441,444^

Additionally, the proteasome-associated DUB USP14 is also well known for suppressing substrate degradation by separating proteasome-bound polyubiquitin chains.^445^ USP14 works as an oncogene and is overexpressed in several cancers, which may be associated with WNT/β-catenin signaling.^446,447^ Moreover, USP14 is also positively correlated with tumor recurrence and poor prognosis.^448,449^ After chemical library screening, the inhibitor IU1 was identified to bind specifically to the activated form of USP14 and abrogate its enzymatic activity (Table 1). Further high-resolution cocrystal structure analysis revealed that IU1 and its analogs can bind to a previously unknown steric binding site in USP14 (ref. ^450^) and enhance proteasome function by blocking the access of the C-terminus of Ub to the active site of USP14.^451^

Currently, many new screening methods have been developed and used to select small-molecule inhibitors and compounds for DUB. For example, high-throughput screening is used to identify small-molecule inhibitors selectively targeting Ub C-terminal hydrolase (UCH-L1). A class of isatin O-acyl oximes (LDN-57444) were found and shown to induce apoptotic cell death in lung tumor cell lines (Fig. 5).^452^ Due to the limited aqueous solubility of LDN-57444, its soluble form, LDN-Pox, was then developed and proved to have the potential to treat invasive carcinomas, including EBV-positive malignancies.^453^ In addition, a cell-based screening was also used to select compounds inducing cathepsin-dependent apoptosis. Intriguingly, b-AP15 was identified to induce the accumulation of high-molecular-mass Ub complexes in cells (Fig. 5).^454^ It is a 19S regulatory particle inhibitor that selectively inhibits the deubiquitinating activity of USP14 and UCHL5 without inhibiting the proteasome activity.^455^ Additionally, it can also block the degradation of a proteasome-degraded reporter protein, resulting in the accumulation of polyubiquitin and inducing strong proteotoxic stress and mitochondrial damage.^456,457^ In many solid tumors and MM, b-AP15 can induce tumor cell apoptosis, which may be associated with c-Myc-Noxa-mediated apoptosis.^455,456,458^

### Multitarget combination treatment

Drug adverse effects and resistance are major obstacles in preclinical and clinical cancer treatments, and UPS inhibitors are no exception.^459–461^ For drug adverse effects, the balance between effective dose and dose limiting toxicity is the principal contradiction.^462^ The molecular mechanisms of anti-cancer drug resistance are also associated with tumor metabolism, the TME and CSCs, such as increasing drug metabolism and degradation of drug target proteins, enhance the tolerability of stressful TME conditions, and enhance the DNA damage response and anti-apoptotic mechanisms of CSCs.^461,463–466^ Thus, to improve therapeutic effects, a multitargeted combination treatment is proposed for UPS inhibitors.

Due to the good curative effects of PIs in MM, a monotherapy of PIs has become the standard of care for patients with MM.^467^ In regard to relapsed MM, recommended therapies usually involve PIs and immunomodulatory drugs (IMiDs; e.g., lenalidomide, pomalidomide) in doublet or triplet combinations with corticosteroids or other systemic therapies including the anti-CD38 monoclonal antibody daratumumab and the immunoglobulin G1 (IgG1) monoclonal antibody isatuximab for the CD38 receptor.^468–470^ For patients with MCL and diffuse large B-cell lymphoma, combination treatment of PIs and chemotherapies or histone deacetylase inhibitors also yields benefits,^471,472^ which may also overcome the impact of gain-of-function p53 mutations in solid tumors.^473^ In the clinic, with regard to bortezomib-resistant tumors, the combination treatments of bortezomib with chemotherapy drugs such as doxorubicin, plerixafor and daratumumab have shown improved clinical outcomes, suggesting that conventional chemotherapy could increase the sensitivity of bortezomib to malignancies.^474–476^ Moreover, combined treatment is also very common for carfilzomib. Combined with lenalidomide and dexamethasone, carfilzomib achieves a near complete clinical response in naive MM patients.^477^ In patients with relapsed/refractory MM, carfilzomib and dexamethasone (Kd56) demonstrate a longer PFS than that of bortezomib and dexamethasone (Vd).^478^ Panobinostat, an HDAC inhibitor, is also used with carfilzomib in MM patients and achieves a good response rate (ClinicalTrial.gov: NCT01549431).^359^

In addition to combination treatment targeting UPS and other signaling pathways, combined inhibitors within the UPS also work in cancer treatment. In PI-resistant MM, inhibiting upstream components of UPS is a promising interest. For example, high expression of USP7 is found in bortezomib-resistant MM and is associated with a short overall survival and poor outcome. As a preclinical practice, the usage of USP7 inhibitors combined with bortezomib triggers synergistic antitumor activity.^479^ Additionally, in PI-resistant MM, the E1 Ub-activating enzyme inhibitor TAK-243 can also block myeloma cell proliferation and induce apoptosis.^480^

## Conclusions and future perspectives

The UPS, a network of enzymes, has been researched for nearly 40 years since its first discovery in 1975. As an important PTM, ubiquitination can regulate a large number of signaling pathways and take part in many biological processes. E3 ligases, regarded as the “brain” in the UPS, can select a specific E2 and substrate and directly transfer Ub to the substrate.^481,482^ However, the results obtained from current studies have not explained how E3 recognizes a specific E2 and selects a substrate, along with the selectivity mechanism of specific lysines in the substrate. It will be necessary to explain the mechanisms underlying the regulation of E3s via structure–function studies.

In ubiquitination, an E3 ligase can mediate divergent functions of substrates by regulating different types of ubiquitination.^6^ SKP2 can not only regulate the stability of c-Myc by promoting its K48 linkage polyubiquitination but also control the activity of AKT/RagA/LKB instead of its stability by mediating K63 linkage polyubiquitination.^131,159,176^ A similar situation occurs with Mdm2, which can promote both the K48 linkage ubiquitination and the neddylation of p53.^219,483^ To understand the dynamics and complexity of such events, it will be necessary to place special emphasis on dissecting the diverse mechanisms of Ub chain assembly by E3s.

In addition to classical ubiquitination, nonclassical ubiquitination, including ubiquitination mediated by SidE that does not depend on E1 and E2,^93,94^ and nonclassical ubiquitination sites (Ser/Thr/Cys) via the formation of thio- or hydroxy-bond esters, are also important parts of ubiquitination.^484–492^ Although many efforts have been made, there are still some unresolved problems in the UPS. For example, although K48- and K11-type ubiquitination can serve as a degradation signal for transferring the target to the 26S proteasome,^37,493^ it is still unclear how the proteasome recognizes K11-type ubiquitination and distinguishes different types of ubiquitination. In addition, the length of the Ub chain is also an open question. There is an urgent need to identify the length of the Ub chain in cells and its befitting length in regulating the function of the substrate. Moreover, it has been reported that the K48-type Ub chain consisting of four Ubs can be degraded by the proteasome.^494,495^ Why four Ubs? These lengths of the Ub chain are also required for other linkage Ub chains. The existence of these problems is closely related to the technical deficiency in detecting the length of the polyubiquitin chain; thus, useful technology to address the looming question is the key to unlocking new areas of UPS.

Because of the importance of the UPS in normal biological processes, its alterations often contribute to the etiology of many diseases, particularly cancer.^3^ The roles of the UPS in tumorigenesis are not only associated with tumor metabolism regulation (mTORC1/AMPK/AKT) but also related to immunological TME modulation (TLR/RLR/STING) and CSC stemness maintenance (Nanog/Oct4/Sox2/Hippo/Wnt).^496–499^ As targeting the TME is a hotspot in the field of cancer treatment, many studies have reported the roles of ubiquitination in tumor immunology. For example, the stability of PD-L1 is regulated by SPOP via proteasome-mediated degradation in cancer cells.^500^ FBXO38, an E3 ligase of PD-1, mediates K48-linked polyubiquitination and subsequent proteasome degradation in activated T cells.^501^ Additionally, COP9 signalosome 5 (CSN5) is required for PD-L1 stabilization by inhibiting its ubiquitination and degradation in cancer cells.^502^ However, the specific role of ubiquitination in T cells, macrophages and DC cells is still unclear, especially for immunotherapies targeting ubiquitination, and need further development.

Based on aberrant UPS activity frequently observed in human cancers, potential therapeutic targets have been identified, and corresponding inhibitors have been developed.^11,65,503^ Currently, the proteasome is a successful target in the clinic, and good therapeutic results are achieved for some FDA-approved PIs, such as bortezomib, carfilzomib, oprozomib and ixazomib. However, as the last step of the ubiquitination process, these PIs can result in some side effects due to the accumulation of upstream ubiquitinated proteins, which limits their widespread application. Thus, attempts have been made to explore targeted inhibitors for E2, E3 ligases, the UBL system and the process of UBD binding to Ub.^504,505^ However, most of these inhibitors work well in cell culture studies and not well enough in animal models and clinical trials.^164,168,506^ One reason for this unsatisfactory situation is the incomplete understanding of the structural analysis of target proteins, pharmaceutical chemistry and combinatorial chemistry, which needs the advances in technology. In addition, high-throughput screening may also help to identify the most feasible inhibitors. Moreover, aberrant activity of the UPS along with other oncogenic signaling pathways may occur simultaneously during the process of tumorigenesis, which makes targeted therapies more complicated. Thus, multitarget combination treatment is recommended as a future direction. Moreover, genomics and proteomics studies based on a large number of patient tumor tissue samples should be adopted to better understand the dynamic process of tumorigenesis. In the end, in-depth exploration of the functions of the UPS and conducting more clinical studies are needed to elucidate the roles of the UPS in tumorigenesis and to develop novel strategies for the treatment and prevention of human cancers.
